# Nutritional Risk Index Predicts Survival in Patients With Breast Cancer Treated With Neoadjuvant Chemotherapy

**DOI:** 10.3389/fnut.2021.786742

**Published:** 2022-01-13

**Authors:** Li Chen, Yihang Qi, Xiangyi Kong, Zhaohui Su, Zhongzhao Wang, Xiangyu Wang, Yaying Du, Yi Fang, Xingrui Li, Jing Wang

**Affiliations:** ^1^Department of Thyroid and Breast Surgery, Tongji Hospital, Tongji Medical College of Huazhong University of Science and Technology, Wuhan, China; ^2^Department of Breast Surgical Oncology, National Cancer Center/National Clinical Research Center for Cancer/Cancer Hospital, Chinese Academy of Medical Sciences and Peking Union Medical College, Beijing, China; ^3^Center on Smart and Connected Health Technologies, Mays Cancer Center, School of Nursing, University of Texas Health Science Center, San Antonio, TX, United States

**Keywords:** nutritional risk index, breast cancer, nutrition, neoadjuvant chemotherapy, prognosis

## Abstract

Nutritional risk index (NRI) is an index based on ideal body weight that aims to present body weight and serum albumin levels. It has been utilized to discriminate patients at risk of postoperative complications and predict the postoperative outcome of major surgeries. However, this index remains limited for breast cancer patients treated with neoadjuvant chemotherapy (NACT). The research explores the clinical and prognostic significance of NRI in breast cancer patients. This study included 785 breast cancer patients (477 cases received NACT and 308 cases did not) were enrolled in this retrospective study. The optimal NRI cutoff value was evaluated by receiver operating characteristic (ROC) curve, then reclassified as low NRI group (<112) and high NRI group (≥112). The results demonstrated that NRI independently predicted survival on disease-free survival (DFS) and overall survival (OS) by univariate and multivariate Cox regression survival analyses [*P* = 0.019, hazard ratio (HR): 1.521, 95% CI: 1.071–2.161 and *P* = 0.004, HR: 1.415, 95% CI: 1.119–1.789; and *P* = 0.026, HR:1.500, 95% CI: 1.051–2.143 and *P* < 0.001, HR: 1.547, 95% CI: 1.221–1.959]. According to the optimal cutoff value of NRI, the high NRI value patients had longer mean DFS and OS time in contrast to those with low NRI value patients (63.47 vs. 40.50 months; 71.50 vs. 56.39 months). Furthermore, the results demonstrated that the high NRI score patients had significantly longer mean DFS and OS time than those with low NRI score patients in early-stage breast cancer (χ^2^ = 9.0510, *P* = 0.0026 and χ^2^ = 9.2140, *P* = 0.0024) and advanced breast cancer (χ^2^ = 6.2500, *P* = 0.0124 and χ^2^ = 5.8880, *P* = 0.0152). The mean DFS and OS values in patients with high NRI scores were significantly longer in contrast to those with low NRI scores in different molecular subtypes. The common toxicities after NACT were hematologic and gastrointestinal reactions, and the NRI had no statistically significant effects on toxicities, except in nausea (χ^2^ = 9.2413, *P* = 0.0024), mouth ulcers (χ^2^ = 4.8133, *P* = 0.0282), anemia (χ^2^ = 8.5441, *P* = 0.0140), and leukopenia (χ^2^ = 11.0951, *P* = 0.0039). NRI serves as a minimally invasive, easily accessible and convenient prognostic tool for evaluating breast cancer prognoses and treatment efficacy, and may help doctors in terms of selecting measures of greater efficiency or appropriateness to better treat breast cancer.

## Introduction

Breast cancer is among the most frequently diagnosed cancers in women globally, and seriously endangers their health (1). Although breast cancer often yields relatively more satisfactory prognoses compared to other types of cancer (e.g., lung cancer), the survival outcomes of patients with aggressive pathological breast cancer or distant metastasis remain to be alarmingly poor—about 90% of breast cancer deaths are caused by the occurrence of distant metastasis (2). As scientific evidence accumulates, treatment strategies, such as surgery, hormone therapy, targeted therapy, and immunotherapy, have forged a comprehensive network of promising treatments with varying degrees of curative effects (3). Aside from the differences in disease conditions, nutritional status also plays an essential role in shaping patients' prognosis as well as treatment efficacy and outcomes.

Decreased appetite with weight loss and cachexia, for instance, can be commonly found in cancer patients (4, 5). As a complicated and multifactorial syndrome, cachexia affects ~50–80% of cancer patients, and is correlated with 20–40% of cancer deaths (6). It is important to note that poor nutritional status not only accelerates the progression of cancer, but also hinders the treatment of the disease, effectively creating a vicious circle that impacts both cancer care and treatment (7, 8). Previous studies found that malnutrition could cause patients' poor response to antitumor therapy, increase the incidence of postoperative complications, and subsequently, result in unsatisfactory survival prognosis (9, 10). In addition, cachexia may be a direct cause of death for cancer patients (11). In one retrospective autopsy study, for instance, the results show that ~1% of 486 patients with cancer died from no other cause but cachexia (11). While some emerging evidence suggests that response rates of chemotherapy were lower among weight-losing patients, limited research on this relationship in breast cancer patients is available (12). Hence, it is of vital significance to discover more convenient indicators to evaluate the effect of nutritional status on disease prognosis and treatment efficacy in breast cancer patients.

Currently known indicators that reflect patients' nutritional status range from the assessment of patients' total body weight (TBW), globulin (GLB), albumin to globulin ratio (AGR), body mass index (BMI), to the prognostic nutritional index (PNI). For instance, previous studies show that malnutrition was related to poor treatment outcomes among patients with various types of cancers (13–15). Nevertheless, people know little about the relationship between nutritional status, cancer prognosis, and treatment efficacy in breast cancer patients (16). Existing evidence often suggests that breast cancer might be related to overnutrition, as opposed to malnutrition (17), effectively contradicting what is known about the predictive role of nutritional status in cancer patients.

To further cloud the research field, research indicates that factors such as BMI might be an unstable indicator of breast cancer patients' nutrition status-the relationship between BMI and the risk of women developing breast cancer differs by patients' menopausal status: in premenopausal women, most studies found either no association or a weak inverse correlation (18); however, in postmenopausal women, greater levels of BMI often increase women's likelihood of receiving a breast cancer diagnosis (19). One way to better shed light on the relationship between nutritional status, cancer prognosis, and treatment efficacy in breast cancer patients is via close examinations of less-studied factors such as the Nutritional Risk Index (NRI).

NRI is one of the most promising assessment tools in gauging the impact of nutritional status on cancer patients' morbidity and mortality rates (20). It is a composite index that factors in changes in patients' ideal body weight, present body weight, and serum albumin levels, and could serve as a convenient screening mechanism to predict the incidence rate of nutrition-related morbidity and mortality in cancer patients (21). For instance, current evidence suggests that low preoperative NRI was associated with poor prognosis and increased postoperative complications and can serve as an indicator in elderly colorectal cancer patients (22). However, this index remains limited for breast cancer patients treated with neoadjuvant chemotherapy. Therefore, to bridge the research gap, the current study aims to evaluate the clinical and prognostic significance of NRI in breast cancer patients, and the correlation between NRI and the treatment efficacy.

## Materials and Methods

### Study Population

The retrospective study included a total of 785 participants-477 patients with breast cancer undergoing NACT (NACT group) and 308 breast cancer patients as control (non-NACT group). All patients received surgery at a large national hospital located in Beijing, China between January 1998 and December 2016. Anthracyclines-based and/or taxanes-based chemotherapy regimens were used for 477 breast cancer patients received NACT treatment. The detailed clinicopathological data were obtained from the patients' electronic medical records. This study was covered under Institutional Review Board (IRB) approved of Cancer Hospital Chinese Academy of Medical Sciences and Tongji Hospital, and it adheres to the standards of the Declaration of Helsinki and its subsequent amendments. All of the patients provided written consent before participating in the study.

Participants were considered as eligible if they were breast cancer patients who had: (1) Confirmed by pathology; (2) Undergone primary tumor resection; (3) Performance Status (Zubrod-ECOG-WHO, ZPS) between 0 and 2 scores, and Karnofsky Performance Scores (KPS) ≥80 scores; (4) complete clinical recorded and follow-up data for all patients; (5) Expected to survive over 3 months; (6) Admission examination showed no obvious abnormalities in liver and renal function. Exclusion criteria were: (1) Patients received relevant anti-tumor therapy, such as chemotherapy, radiotherapy; (2) With serious complications, for instance, infection, pneumonia, skin ulcer; (3) Patients with chronic inflammatory diseases or autoimmune disease, for example, liver cirrhosis, systemic lupus erythematosus (SLE); (4) With distant organ metastasis; (5) Blood product transfusion within 1 month before treatment.

### Pre-treatment Evaluation and TNM Classification

The 8th edition American Joint Committee on Cancer (AJCC) and the Union for International Cancer Control (UICC) were used to evaluate TNM stage classification (23, 24). The Response Evaluation Criteria in Solid Tumors (RECIST) guidelines were performed to evaluate the response rates of patients who received NACT (25). The Miller and Payne grade (MPG) framework was used to assess the histological response of the participants (26). The National Cancer Institute Common Toxicity Criteria (NCI-CTC) was used to assess the chemotherapy toxicity and adverse effects (27). Molecular classification of breast cancer was triple-negative type, HER2-enriched type, Luminal B HER2-negative type, Luminal B HER2-positive type, and Luminal A type, respectively (28).

### Peripheral Venous Blood Parameters and Nutritional Factors

All of patients' blood samples were taken within 7 days before treatment. NRI is calculated as follows: 1.519 × serum albumin level (g/l) + 41.7 × (present/ideal body weight). And the ideal weight (Wlo) was calculated using the following formula: Height-100-[(Height-150)/2.5].

### Follow-Up

Follow-up modalities included clinical examination, laboratory tests (routine blood test and blood biochemical), imaging examination (ultrasonography, mammography, and computed tomography of the chest). Follow-up evaluations were performed: (1) every 3 months for the first to second year postoperatively, (2) every 6 months for the third to fifth year postoperatively, (3) then yearly thereafter. Disease-free survival (DFS) was the duration from date of surgery to tumor recurrence, distant metastases, the date of death from any cause or last follow-up. Overall survival (OS) was the duration from the date of surgery to the date of death from any cause or last follow-up. Follow-up data were obtained from medical records, both inpatients and outpatients.

### Statistical Analysis

The optimal cutoff values of related variables were utilized receiver operating characteristic (ROC) curves. The qualitative data was presented as the number of cases (%), and with intergroup comparisons performed in Chi-square test or Fisher's exact test. Survival curves, including DFS and OS, were generated using the Kaplan-Meier method coupled with the Log-rank test. The univariate and multivariate Cox proportional hazards regression model was used to discern potential prognostic factors. The association between patients' NRI and prognosis was performed using hazard ratios (HRs) and 95% confidence intervals (CIs). All statistical analyses were carried out by SPSS 17.0 (SPSS Inc., Chicago, IL, USA) and GraphPad prism 8.0 (GraphPad Inc., La Jolla, CA, USA). Alpha was set at the 0.05 level, and a two-tailed *P* < 0.05 was interpreted to achieve statistically significant.

## Results

### Demographic and Clinicopathologic Features

The ROC curve was used to confirm the optimal cutoff value of NRI, and the value was 112. Two NRI groups were formed by the optimal NRI cutoff value: low NRI group (NRI <112) and high NRI group (NRI ≥ 112). Of all patients, in the results demonstrated that age (χ^2^ = 4.2272, *P* = 0.0398), menopause (χ^2^ = 12.6300, *P* = 0.0004), US-LNM (χ^2^ = 6.6599, *P* = 0.0099), total lymph nodes (χ^2^ = 8.7863, *P* = 0.0030), total axillary lymph nodes (χ^2^ = 6.9193, *P* = 0.0085) were statistically significant differences between the two NRI groups. Other parameters were not statistically significant differences between the two NRI groups (*P* > 0.05) (see Table 1).

**Table 1:** Demographic and clinicopathologic characteristics of the study's 785 breast cancer participants.

**Parameters**	**NRI 785**				**NRI 477**				**NRI 308**			
**Cases (*n*)**	**Low NRI 291**	**High NRI 494**	**χ^2^**	***P*-value**	**Low NRI 174**	**High NRI 303**	**χ^2^**	***P*-value**	**Low NRI 117**	**High NRI 191**	**χ^2^**	***P*-value**
Age (years)			4.2272	0.0398			7.2047	0.0073			0.0037	0.9514
<47	157 (53.95%)	229 (46.36%)			98 (56.32%)	132 (43.56%)			59 (50.43%)	97 (50.79%)		
≥47	134 (46.05%)	265 (53.64%)			76 (43.68%)	171 (56.44%)			58 (49.57%)	94 (49.21%)		
Family history			0.5565	0.4557			3.3583	0.0669			1.4663	0.2259
No	217 (74.57%)	380 (76.92%)			118 (67.82%)	229 (75.58%)			99 (84.62%)	151 (79.06%)		
Yes	74 (25.43%)	114 (23.08%)			56 (32.18%)	74 (24.42%)			18 (15.38%)	40 (20.94%)		
Menopause			12.6300	0.0004			8.2428	0.0041			4.2263	0.0398
No	206 (70.79%)	287 (58.10%)			117 (67.24%)	163 (53.80%)			89 (76.07%)	124 (64.92%)		
Yes	85 (29.21%)	207 (41.90%)			57 (32.76%)	140 (46.20%)			28 (23.93%)	67 (35.08%)		
ABO blood type			0.3976	0.9827			2.0368	0.7290			1.8269	0.7676
A	76 (26.12%)	138 (27.94%)			42 (24.14%)	90 (29.70%)			34 (29.06%)	48 (25.13%)		
B	97 (33.33%)	165 (33.40%)			58 (33.33%)	87 (28.71%)			39 (33.33%)	78 (40.84%)		
O	89 (30.58%)	145 (29.35%)			54 (31.03%)	92 (30.36%)			35 (29.91%)	53 (27.75%)		
AB	29 (9.97%)	46 (9.31%)			20 (11.49%)	34 (11.22%)			9 (7.69%)	12 (6.28%)		
Tumor site			0.8458	0.3578			0.0358	0.8500			3.0094	0.0828
Right	143 (49.14%)	226 (45.75%)			84 (48.28%)	149 (49.17%)			59 (50.43%)	77 (40.31%)		
Left	148 (50.86%)	268 (54.25%)			90 (51.72%)	154 (50.83%)			58 (49.57%)	114 (59.69%)		
US-Primary tumor site			5.1400	0.2732			6.7210	0.1514			3.3700	0.4979
Upper outer quadrant	190 (65.29%)	299 (60.53%)			116 (66.67%)	189 (62.38%)			74 (63.25%)	110 (57.59%)		
Lower outer quadrant	21 (7.22%)	60 (12.15%)			9 (5.17%)	35 (11.55%)			12 (10.26%)	25 (13.09%)		
Lower inner quadrant	13 (4.47%)	24 (4.86%)			9 (5.17%)	9 (2.97%)			4 (3.42%)	15 (7.85%)		
Upper inner quadrant	46 (15.81%)	74 (14.98%)			23 (13.22%)	38 (12.54%)			23 (19.66%)	36 (18.85%)		
Central	21 (7.22%)	37 (7.49%)			17 (9.77%)	32 (10.56%)			4 (3.42%)	5 (2.62%)		
US-Tumor size (cm)			3.5999	0.1653			3.0109	0.2219			1.7944	0.4077
≤ 2cm	105 (36.08%)	205 (41.50%)			44 (25.29%)	91 (30.03%)			61 (52.14%)	114 (59.69%)		
>2 and <5 cm	153 (52.58%)	249 (50.40%)			99 (56.90%)	174 (57.43%)			54 (46.15%)	75 (39.27%)		
≥5 cm	33 (11.34%)	40 (8.10%)			31 (17.82%)	38 (12.54%)			2 (1.71%)	2 (1.05%)		
US-LNM			6.6599	0.0099			4.3998	0.0359			2.1557	0.1421
No	230 (79.04%)	349 (70.65%)			125 (71.84%)	189 (62.38%)			105 (89.74%)	160 (83.77%)		
Yes	61 (20.96%)	145 (29.35%)			49 (28.16%)	114 (37.62%)			12 (10.26%)	31 (16.23%)		
US-BIRADS			0.2781	0.8702			0.7660	0.6818			0.2191	0.8963
4	27 (9.28%)	51 (10.32%)			18 (10.34%)	36 (11.88%)			9 (7.69%)	15 (7.85%)		
5	118 (40.55%)	202 (40.89%)			64 (36.78%)	119 (39.27%)			54 (46.15%)	83 (43.46%)		
6	146 (50.17%)	241 (48.79%)			92 (52.87%)	148 (48.84%)			54 (46.15%)	93 (48.69%)		
Clinical T stage			1.1766	0.8819			0.7925	0.9395			2.3854	0.6653
T1	59 (20.27%)	109 (22.06%)			25 (14.37%)	40 (13.20%)			34 (29.06%)	69 (36.13%)		
T2	154 (52.92%)	259 (52.43%)			80 (45.98%)	146 (48.18%)			74 (63.25%)	113 (59.16%)		
T3	53 (18.21%)	78 (15.79%)			45 (25.86%)	70 (23.10%)			8 (6.84%)	8 (4.19%)		
T4	25 (8.59%)	48 (9.72%)			24 (13.79%)	47 (15.51%)			1 (0.85%)	1 (0.52%)		
Clinical N stage			6.8947	0.1416			3.2495	0.5170			4.8157	0.3067
N0	125 (42.96%)	174 (35.22%)			31 (17.82%)	42 (13.86%)			94 (80.34%)	132 (69.11%)		
N1	75 (25.77%)	158 (31.98%)			56 (32.18%)	108 (35.64%)			19 (16.24%)	50 (26.18%)		
N2	53 (18.21%)	107 (21.66%)			50 (28.74%)	101 (33.33%)			3 (2.56%)	6 (3.14%)		
N3	38 (13.06%)	55 (11.13%)			37 (21.26%)	52 (17.16%)			1 (0.85%)	3 (1.57%)		
Clinical TNM stage			1.0040	0.6053			0.6262	0.7312			0.5983	0.7415
I	34 (11.68%)	58 (11.74%)			6 (3.45%)	8 (2.64%)			28 (23.93%)	50 (26.18%)		
II	148 (50.86%)	234 (47.37%)			64 (36.78%)	104 (34.32%)			84 (71.79%)	130 (68.06%)		
III	109 (37.46%)	202 (40.89%)			104 (59.77%)	191 (63.04%)			5 (4.27%)	11 (5.76%)		
Neoadjuvant Chemotherapy (PRE)							3.9810	0.4085				
AC/ACF					6 (3.45%)	22 (7.26%)						
CT/ACT					11 (6.32%)	16 (5.28%)						
AT					86 (49.43%)	137 (45.21%)						
TP					48 (27.59%)	93 (30.69%)						
Others					23 (13.22%)	35 (11.55%)						
Chemotherapy times (PRE)							0.4359	0.5091				
<6					52 (29.89%)	82 (27.06%)						
≥6					122 (70.11%)	221 (72.94%)						
Response							4.0382	0.4009				
CR					3 (1.72%)	4 (1.32%)						
PR					110 (63.22%)	202 (66.67%)						
SD					56 (32.18%)	95 (31.35%)						
PD					5 (2.87%)	2 (0.66%)						
Miller and Payne grade							5.3440	0.2538				
1					7 (4.02%)	15 (4.95%)						
2					40 (22.99%)	86 (28.38%)						
3					63 (36.21%)	114 (37.62%)						
4					30 (17.24%)	32 (10.56%)						
5					34 (19.54%)	56 (18.48%)						
Pathological response							0.0382	0.8450				
pCR					27 (15.52%)	45 (14.85%)						
non-pCR					147 (84.48%)	258 (85.15%)						
Post-chemotherapy regimen			0.9129	0.9693			2.5610	0.7673			2.9160	0.7129
AC/ACF	47 (16.15%)	78 (15.79%)			13 (7.47%)	30 (9.90%)			34 (29.06%)	48 (25.13%)		
CT/ACT	48 (16.49%)	77 (15.59%)			12 (6.90%)	18 (5.94%)			36 (30.77%)	59 (30.89%)		
AT	38 (13.06%)	59 (11.94%)			17 (9.77%)	20 (6.60%)			21 (17.95%)	39 (20.42%)		
TP	24 (8.25%)	37 (7.49%)			15 (8.62%)	24 (7.92%)			9 (7.69%)	13 (6.81%)		
Others	37 (12.71%)	71 (14.37%)			30 (17.24%)	51 (16.83%)			7 (5.98%)	20 (10.47%)		
NO	97 (33.33%)	172 (34.82%)			87 (50.00%)	160 (52.81%)			10 (8.55%)	12 (6.28%)		
Operative time (min)			0.7026	0.4019			0.1904	0.6626			0.4766	0.4900
<90	123 (42.27%)	224 (45.34%)			90 (51.72%)	163 (53.80%)			33 (28.21%)	61 (31.94%)		
≥90	168 (57.73%)	270 (54.66%)			84 (48.28%)	140 (46.20%)			84 (71.79%)	130 (68.06%)		
Type of surgery			0.4121	0.5209			2.6578	0.1030			0.5543	0.4566
Mastectomy	221 (75.95%)	385 (77.94%)			142 (81.61%)	264 (87.13%)			79 (67.52%)	121 (63.35%)		
Breast-conserving surgery	70 (24.05%)	109 (22.06%)			32 (18.39%)	39 (12.87%)			38 (32.48%)	70 (36.65%)		
Tumor size			0.6829	0.7108			1.4411	0.4865			8.8906	0.0117
≤ 2 cm	157 (53.95%)	280 (56.68%)			102 (58.62%)	161 (53.14%)			55 (47.01%)	119 (62.30%)		
>2 and <5 cm	114 (39.18%)	185 (37.45%)			57 (32.76%)	115 (37.95%)			57 (48.72%)	70 (36.65%)		
≥5 cm	20 (6.87%)	29 (5.87%)			15 (8.62%)	27 (8.91%)			5 (4.27%)	2 (1.05%)		
Histologic type			1.7407	0.4188			4.1249	0.1271			0.3858	0.8246
Ductal	284 (97.59%)	474 (95.95%)			172 (98.85%)	289 (95.38%)			112 (95.73%)	185 (96.86%)		
Lobular	4 (1.37%)	9 (1.82%)			1 (0.57%)	6 (1.98%)			3 (2.56%)	3 (1.57%)		
Others	3 (1.03%)	11 (2.23%)			1 (0.57%)	8 (2.64%)			2 (1.71%)	3 (1.57%)		
Histologic grade			1.3423	0.5111			3.0411	0.2186			13.3849	0.0012
I	52 (17.87%)	81 (16.40%)			34 (19.54%)	74 (24.42%)			18 (15.38%)	7 (3.66%)		
II	164 (56.36%)	267 (54.05%)			98 (56.32%)	146 (48.18%)			66 (56.41%)	121 (63.35%)		
III	75 (25.77%)	146 (29.55%)			42 (24.14%)	83 (27.39%)			33 (28.21%)	63 (32.98%)		
Pathological T stage			2.5200	0.6411			5.7720	0.2169			4.1800	0.3822
Tis/T0	35 (12.03%)	57 (11.54%)			32 (18.39%)	56 (18.48%)			3 (2.56%)	1 (0.52%)		
T1	113 (38.83%)	189 (38.26%)			76 (43.68%)	114 (37.62%)			37 (31.62%)	75 (39.27%)		
T2	114 (39.18%)	212 (42.91%)			44 (25.29%)	105 (34.65%)			70 (59.83%)	107 (56.02%)		
T3	21 (7.22%)	24 (4.86%)			16 (9.20%)	18 (5.94%)			5 (4.27%)	6 (3.14%)		
T4	8 (2.75%)	12 (2.43%)			6 (3.45%)	10 (3.30%)			2 (1.71%)	2 (1.05%)		
Pathological N stage			3.2307	0.5200			2.0263	0.7309			6.1693	0.1869
N0	124 (42.61%)	202 (40.89%)			67 (38.51%)	109 (35.97%)			57 (48.72%)	93 (48.69%)		
N1	56 (19.24%)	119 (24.09%)			35 (20.11%)	66 (21.78%)			21 (17.95%)	53 (27.75%)		
N2	51 (17.53%)	71 (14.37%)			32 (18.39%)	45 (14.85%)			19 (16.24%)	26 (13.61%)		
N3	60 (20.62%)	102 (20.65%)			40 (22.99%)	83 (27.39%)			20 (17.09%)	19 (9.95%)		
Pathological TNM stage			2.8211	0.5882			5.8386	0.2115			3.7345	0.4431
Tis/T0	28 (9.62%)	46 (9.31%)			26 (14.94%)	45 (14.85%)			2 (1.71%)	1 (0.52%)		
I	64 (21.99%)	93 (18.83%)			39 (22.41%)	44 (14.52%)			25 (21.37%)	49 (25.65%)		
II	87 (29.90%)	175 (35.43%)			36 (20.69%)	82 (27.06%)			51 (43.59%)	93 (48.69%)		
III	112 (38.49%)	180 (36.44%)			73 (41.95%)	132 (43.56%)			39 (33.33%)	48 (25.13%)		
Total lymph nodes			8.7863	0.0030			3.9425	0.0471			4.9253	0.0265
<21	165 (56.70%)	226 (45.75%)			84 (48.28%)	118 (38.94%)			81 (69.23%)	108 (56.54%)		
≥21	126 (43.30%)	268 (54.25%)			90 (51.72%)	185 (61.06%)			36 (30.77%)	83 (43.46%)		
Positive lymph nodes			0.3660	0.5452			0.5296	0.4668			0.0127	0.9101
<1	126 (43.30%)	203 (41.09%)			69 (39.66%)	110 (36.30%)			57 (48.72%)	93 (48.69%)		
≥1	165 (56.70%)	291 (58.91%)			105 (60.34%)	193 (63.70%)			60 (51.28%)	98 (51.31%)		
Total axillary lymph nodes			6.9193	0.0085			5.2727	0.0217			1.6639	0.1971
<20	162 (55.67%)	227 (45.95%)			83 (47.70%)	112 (36.96%)			79 (67.52%)	115 (60.21%)		
≥20	129 (44.33%)	267 (54.05%)			91 (52.30%)	191 (63.04%)			38 (32.48%)	76 (39.79%)		
Positive axillary lymph nodes			0.0160	0.8993			0.2612	0.6093			0.2575	0.6118
<1	128 (43.99%)	215 (43.52%)			69 (39.66%)	113 (37.29%)			59 (50.43%)	102 (53.40%)		
≥1	163 (56.01%)	279 (56.48%)			105 (60.34%)	190 (62.71%)			58 (49.57%)	89 (46.60%)		
Post-operative complications			0.7944	0.3728			4.4512	0.0349			0.6359	0.4252
No	273 (93.81%)	455 (92.11%)			169 (97.13%)	280 (92.41%)			104 (88.89%)	175 (91.62%)		
Yes	18 (6.19%)	39 (7.89%)			5 (2.87%)	23 (7.59%)			13 (11.11%)	16 (8.38%)		
Post-operative chemotherapy			0.1792	0.6721			0.3484	0.5550			0.5609	0.4539
No	97 (33.33%)	172 (34.82%)			87 (50.00%)	160 (52.81%)			10 (8.55%)	12 (6.28%)		
Yes	194 (66.67%)	322 (65.18%)			87 (50.00%)	143 (47.19%)			107 (91.45%)	179 (93.72%)		
Post-operative chemotherapy times			0.1528	0.6959			0.0100	0.9205			0.1177	0.7316
<4	136 (46.74%)	238 (48.18%)			124 (71.26%)	216 (71.29%)			12 (10.26%)	22 (11.52%)		
≥4	155 (53.26%)	256 (51.82%)			50 (28.74%)	87 (28.71%)			105 (89.74%)	169 (88.48%)		
Post-operative radiotherapy			0.0034	0.9534			0.3244	0.5690			0.3721	0.5419
No	73 (25.09%)	123 (24.90%)			46 (26.44%)	73 (24.09%)			27 (23.08%)	50 (26.18%)		
Yes	218 (74.91%)	371 (75.10%)			128 (73.56%)	230 (75.91%)			90 (76.92%)	141 (73.82%)		
Post-operative endocrine therapy			0.0968	0.7557			0.5481	0.4591			0.1384	0.7099
No	114 (39.18%)	188 (38.06%)			79 (45.40%)	127 (41.91%)			35 (29.91%)	61 (31.94%)		
Yes	177 (60.82%)	306 (61.94%)			95 (54.60%)	176 (58.09%)			82 (70.09%)	130 (68.06%)		
Post-operative targeted therapy			2.3758	0.1232			2.8104	0.0937			0.1659	0.6838
No	207 (71.13%)	376 (76.11%)			113 (64.94%)	219 (72.28%)			94 (80.34%)	157 (82.20%)		
Yes	84 (28.87%)	118 (23.89%)			61 (35.06%)	84 (27.72%)			23 (19.66%)	34 (17.80%)		

### Nutritional Parameters and Hematological Parameters

Of all enrolled patients, there were significant differences in weight (χ^2^ = 165.5080, *P* < 0.0001), Body Mass Index (BMI) (χ^2^ = 189.1500, *P* < 0.0001), Alanine aminotransferase (ALT) (χ^2^ = 14.2711, *P* = 0.0002), Aspartate aminotransferase (AST) (χ^2^ = 8.6402, *P* = 0.0033), Lactate dehydrogenase (LDH) (χ^2^ = 19.1932, *P* < 0.0001), γ-glutamyl transpeptidase (GGT) (χ^2^ = 22.926, *P* < 0.001), Alkaline phosphatase (ALP) (χ^2^ = 12.861, *P* = 0.0003), Blood glucose (GLU) (χ^2^ = 13.713, *P* < 0.001), Immunoglobulin G (IgG) (χ^2^ = 15.8213, *P* < 0.0001), Albumin (ALB) (χ^2^ = 135.2380, *P* < 0.0001), White blood cell (W) (χ^2^ = 6.9193, *P* = 0.0085), Red blood cell (R) (χ^2^ = 34.5983, *P* < 0.0001), Hemoglobin (Hb) (χ^2^=30.5623, *P* < 0.0001), Neutrophil (N) (χ^2^ = 12.2538, *P* = 0.0005), Eosinophils (E) (χ^2^ = 5.6190, *P* = 0.0178), Platelet (P) (χ^2^ = 13.8379, *P* = 0.0002), respectively. The results were shown in Table 2.

**Table 2:** The correlation between nutritional parameters/blood parameters and NRI.

**Parameters**	**NRI 785**				**NRI 477**				**NRI 308**			
**Cases (*n*)**	**Low NRI 291**	**High NRI 494**	**χ^2^**	***P*-value**	**Low NRI 174**	**High NRI 303**	**χ^2^**	***P*-value**	**Low NRI 117**	**High NRI 191**	**χ^2^**	***P*-value**
Weight (Kg)			165.5080	<0.0001			114.6400	<0.0001			52.3078	<0.0001
<62.00	229 (78.69%)	154 (31.17%)			142 (81.61%)	93 (30.69%)			87 (74.36%)	61 (31.94%)		
≥62.00	62 (21.31%)	340 (68.83%)			32 (18.39%)	210 (69.31%)			30 (25.64%)	130 (68.06%)		
Height (m)			0.0191	0.8900			0.2239	0.6361			0.5970	0.4397
<1.60	124 (42.61%)	213 (43.12%)			82 (47.13%)	136 (44.88%)			42 (35.90%)	77 (40.31%)		
≥1.60	167 (57.39%)	281 (56.88%)			92 (52.87%)	167 (55.12%)			75 (64.10%)	114 (59.69%)		
BMI			189.1500	<0.0001			124.4900	<0.0001			65.9453	<0.0001
<24.00	238 (81.79%)	153 (30.97%)			148 (85.06%)	97 (32.01%)			90 (76.92%)	56 (29.32%)		
≥24.00	53 (18.21%)	341 (69.03%)			26 (14.94%)	206 (67.99%)			27 (23.08%)	135 (70.68%)		
ALT (U/L)			14.2711	0.0002			6.3387	0.0118			8.0961	0.0044
<15	163 (56.01%)	207 (41.90%)			89 (51.15%)	119 (39.27%)			74 (63.25%)	88 (46.07%)		
≥15	129 (44.33%)	287 (58.10%)			85 (48.85%)	184 (60.73%)			44 (37.61%)	103 (53.93%)		
AST (U/L)			8.6402	0.0033			4.4634	0.0346			4.0702	0.0437
<18	160 (54.98%)	218 (44.13%)			88 (50.57%)	123 (40.59%)			72 (61.54%)	95 (49.74%)		
≥18	131 (45.02%)	276 (55.87%)			86 (49.43%)	180 (59.41%)			45 (38.46%)	96 (50.26%)		
LDH (U/L)			19.1932	<0.0001			11.6302	0.0007			7.5377	0.0060
<167	169 (58.08%)	207 (41.90%)			88 (50.57%)	105 (34.65%)			81 (69.23%)	102 (53.40%)		
≥167	122 (41.92%)	287 (58.10%)			86 (49.43%)	198 (65.35%)			36 (30.77%)	89 (46.60%)		
GGT (U/L)			22.9262	<0.0001			9.4150	0.0022			14.3058	0.0002
<17	168 (57.73%)	198 (40.08%)			90 (51.72%)	113 (37.29%)			78 (66.67%)	85 (44.50%)		
≥17	123 (42.27%)	296 (59.92%)			84 (48.28%)	190 (62.71%)			39 (33.33%)	106 (55.50%)		
ALP (U/L)			12.8606	0.0003			8.3752	0.0038			4.4880	0.0341
<64	164 (56.36%)	213 (43.12%)			98 (56.32%)	129 (42.57%)			66 (56.41%)	84 (43.98%)		
≥64	127 (43.64%)	281 (56.88%)			76 (43.68%)	174 (57.43%)			51 (43.59%)	107 (56.02%)		
GLU (mmol/L)			13.7133	0.0002			20.6972	<0.0001			0.0934	0.7599
<5.33	170 (58.42%)	221 (44.74%)			114 (65.52%)	133 (43.89%)			56 (47.86%)	88 (46.07%)		
≥5.33	121 (41.58%)	273 (55.26%)			60 (34.48%)	170 (56.11%)			61 (52.14%)	103 (53.93%)		
IgA (g/L)			0.5835	0.4450			0.6877	0.4069			0.0467	0.8289
<2.30	149 (51.20%)	239 (48.38%)			93 (53.45%)	150 (49.50%)			56 (47.86%)	89 (46.60%)		
≥2.30	142 (48.80%)	255 (51.62%)			81 (46.55%)	153 (50.50%)			61 (52.14%)	102 (53.40%)		
IgG (g/L)			15.8213	<0.0001			7.1034	0.0077			9.1460	0.0025
<11.70	170 (58.42%)	216 (43.72%)			99 (56.90%)	134 (44.22%)			71 (60.68%)	82 (42.93%)		
≥11.70	121 (41.58%)	278 (56.28%)			75 (43.10%)	169 (55.78%)			46 (39.32%)	109 (57.07%)		
IgM (g/L)			2.8698	0.0903			1.7770	0.1825			1.0348	0.3090
<1.10	132 (45.36%)	255 (51.62%)			82 (47.13%)	162 (53.47%)			50 (42.74%)	93 (48.69%)		
≥1.10	159 (54.64%)	239 (48.38%)			92 (52.87%)	141 (46.53%)			67 (57.26%)	98 (51.31%)		
ALB (g/L)			135.2380	<0.0001			74.2045	<0.0001			61.3788	<0.0001
<45.2	224 (76.98%)	168 (34.01%)			131 (75.29%)	104 (34.32%)			93 (79.49%)	64 (33.51%)		
≥45.2	67 (23.02%)	326 (65.99%)			43 (24.71%)	199 (65.68%)			24 (20.51%)	127 (66.49%)		
CRP (mg/dl)			0.6978	0.4035			0.0235	0.8783			1.0375	0.3084
<0.02	148 (50.86%)	236 (47.77%)			69 (39.66%)	118 (38.94%)			79 (67.52%)	118 (61.78%)		
≥0.02	143 (49.14%)	258 (52.23%)			105 (60.34%)	185 (61.06%)			38 (32.48%)	73 (38.22%)		
CA125 (U/ml)			2.7964	0.0945			2.1107	0.1463			0.8742	0.3498
<13.35	134 (46.05%)	258 (52.23%)			73 (41.95%)	148 (48.84%)			61 (52.14%)	110 (57.59%)		
≥13.35	157 (53.95%)	236 (47.77%)			101 (58.05%)	155 (51.16%)			56 (47.86%)	81 (42.41%)		
CA153 (U/ml)			0.0620	0.8033			0.3039	0.5814			0.9651	0.3259
<11.63	147 (50.52%)	245 (49.60%)			73 (41.95%)	135 (44.55%)			74 (63.25%)	110 (57.59%)		
≥11.63	144 (49.48%)	249 (50.40%)			101 (58.05%)	168 (55.45%)			43 (36.75%)	81 (42.41%)		
CEA (ng/ml)			0.0378	0.8459			0.0651	0.7986			0.0081	0.9285
<1.66	144 (49.48%)	248 (50.20%)			76 (43.68%)	136 (44.88%)			68 (58.12%)	112 (58.64%)		
≥1.66	147 (50.52%)	246 (49.80%)			98 (56.32%)	167 (55.12%)			49 (41.88%)	79 (41.36%)		
D-D (mg/L)			0.9341	0.3338			0.9454	0.3309			0.0537	0.8167
<0.29	150 (51.55%)	237 (47.98%)			78 (44.83%)	122 (40.26%)			72 (61.54%)	115 (60.21%)		
≥0.29	141 (48.45%)	257 (52.02%)			96 (55.17%)	181 (59.74%)			45 (38.46%)	76 (39.79%)		
FIB (g/L)			1.8362	0.1754			0.6464	0.4214			1.2150	0.2704
<2.85	153 (52.58%)	235 (47.57%)			83 (47.70%)	133 (43.89%)			70 (59.83%)	102 (53.40%)		
≥2.85	138 (47.42%)	259 (52.43%)			91 (52.30%)	170 (56.11%)			47 (40.17%)	89 (46.60%)		
INR			0.1167	0.7326			0.0951	0.7578			0.1161	0.7333
<0.93	133 (45.70%)	232 (46.96%)			63 (36.21%)	114 (37.62%)			70 (59.83%)	118 (61.78%)		
≥0.93	158 (54.30%)	262 (53.04%)			111 (63.79%)	189 (62.38%)			47 (40.17%)	73 (38.22%)		
FDP (ug/ml)			0.2037	0.6518			1.5777	0.2091			0.1936	0.6599
<1.40	133 (45.70%)	234 (47.37%)			44 (25.29%)	93 (30.69%)			89 (76.07%)	141 (73.82%)		
≥1.40	158 (54.30%)	260 (52.63%)			130 (74.71%)	210 (69.31%)			28 (23.93%)	50 (26.18%)		
Before chemotherapy												
White blood cell (W) (×10^9^/L)			6.9193	0.0085			2.2118	0.1370			5.5383	0.0186
<6.01	162 (55.67%)	227 (45.95%)			95 (54.60%)	144 (47.52%)			67 (57.26%)	83 (43.46%)		
≥6.01	129 (44.33%)	267 (54.05%)			79 (45.40%)	159 (52.48%)			50 (42.74%)	108 (56.54%)		
Red blood cell (R) (×10^12^/L)			34.5983	<0.0001			24.9932	<0.0001			10.0475	0.0015
<4.40	184 (63.23%)	205 (41.50%)			112 (64.37%)	123 (40.59%)			72 (61.54%)	82 (42.93%)		
≥4.40	107 (36.77%)	289 (58.50%)			62 (35.63%)	180 (59.41%)			45 (38.46%)	109 (57.07%)		
Hemoglobin (Hb) (×10^9^/L)			30.5623	<0.0001			15.0049	0.0001			16.4623	<0.0001
<132	179 (61.51%)	203 (41.09%)			109 (62.64%)	134 (44.22%)			70 (59.83%)	69 (36.13%)		
≥132	112 (38.49%)	291 (58.91%)			65 (37.36%)	169 (55.78%)			47 (40.17%)	122 (63.87%)		
Neutrophil (N) (×10^9^/L)			12.2538	0.0005			5.6323	0.0176			6.7928	0.0092
<3.68	169 (58.08%)	223 (45.14%)			96 (55.17%)	133 (43.89%)			73 (62.39%)	90 (47.12%)		
≥3.68	122 (41.92%)	271 (54.86%)			78 (44.83%)	170 (56.11%)			44 (37.61%)	101 (52.88%)		
Lymphocyte (L) (×10^9^/L)			0.0043	0.9477			0.3036	0.5816			0.3575	0.5499
<1.76	145 (49.83%)	246 (49.80%)			97 (55.75%)	161 (53.14%)			48 (41.03%)	85 (44.50%)		
≥1.76	146 (50.17%)	248 (50.20%)			77 (44.25%)	142 (46.86%)			69 (58.97%)	106 (55.50%)		
Monocyte (M) (×10^9^/L)			0.1913	0.6619			0.0532	0.8175			0.1483	0.7002
<0.35	139 (47.77%)	228 (46.15%)			80 (45.98%)	136 (44.88%)			59 (50.43%)	92 (48.17%)		
≥0.35	152 (52.23%)	266 (53.85%)			94 (54.02%)	167 (55.12%)			58 (49.57%)	99 (51.83%)		
Eosinophils (E) (×10^9^/L)			5.6190	0.0178			1.2650	0.2607			5.5256	0.0187
<0.06	116 (39.86%)	240 (48.58%)			82 (47.13%)	159 (52.48%)			34 (29.06%)	81 (42.41%)		
≥0.06	175 (60.14%)	254 (51.42%)			92 (52.87%)	144 (47.52%)			83 (70.94%)	110 (57.59%)		
Basophils (B) (×10^9^/L)			2.6581	0.1030			3.1246	0.0771			0.1668	0.6830
<0.02	93 (31.96%)	131 (26.52%)			58 (33.33%)	78 (25.74%)			35 (29.91%)	53 (27.75%)		
≥0.02	198 (68.04%)	363 (73.48%)			116 (66.67%)	225 (74.26%)			82 (70.09%)	138 (72.25%)		
Platelet (P) (×10^9^/L)			13.8379	0.0002			9.6383	0.0019			4.1917	0.0406
<243	169 (58.08%)	219 (44.33%)			98 (56.32%)	126 (41.58%)			71 (60.68%)	93 (48.69%)		
≥243	122 (41.92%)	275 (55.67%)			76 (43.68%)	177 (58.42%)			46 (39.32%)	98 (51.31%)		

### Univariate and Multivariate Cox Regression Survival Analyses for Survival Analysis

The univariate and multivariate Cox proportional-hazards models with time-varying NRI were used to analyze the independent prognostic factors. Through univariate and multivariate Cox regression analysis, menopause, GLU, Cancer antigen 125 (CA125), Cancer antigen 153 (CA153), eosinophils, NRI, histologic type, pathological T/N/TNM stage, Ki-67 status, Cytokeratin 5/6 (CK5/6) status, lymph vessel invasion (LVI), post-operative chemotherapy, post-operative endocrine therapy, post-operative targeted therapy were the significant prognostic factors for DFS. Moreover, GLU, CA153, International normalized ratio (INR), monocyte, eosinophils, NRI, clinical T stage, histologic type, pathological T/N/TNM stage, Ki-67 status, CK5/6 status, E-cadherin (E-cad) status, LVI, post-operative chemotherapy, post-operative endocrine therapy, post-operative targeted therapy were the significant prognostic factors for OS (see Table 3).

**Table 3:** Univariate and multivariate cox regression survival analyses of the NRI for the prediction of DFS and OS in the participants.

	**Univariate analysis**	**DFS**	**Multivariate analysis**		**Univariate analysis**	**OS**	**Multivariate analysis**	
**Parameters**	**Hazard ratio (95% CI)**	***P*** **-value**	**Hazard ratio (95% CI)**	***P*** **-value**	**Hazard ratio (95% CI)**	***P*** **-value**	**Hazard ratio (95% CI)**	***P*** **-value**
Cases (*n*)								
Age (years)		0.6653				0.9316		
<47	1 (reference)				1 (reference)			
≥47	0.926 (0.654–1.311)				1.015 (0.717–1.437)			
Weight (Kg)		0.3371				0.3594		
<62.00	1 (reference)				1 (reference)			
≥62.00	1.212 (0.819–1.793)				1.209 (0.806–1.814)			
Height (m)		0.5863				0.5458		
<1.60	1 (reference)				1 (reference)			
≥1.60	0.926 (0.700–1.223)				0.915 (0.687–1.220)			
BMI		0.0696				0.1769		
<24.00	1 (reference)				1 (reference)			
≥24.00	0.690 (0.462–1.030)				0.754 (0.500–1.136)			
Family history		0.3081				0.7330		
No	1 (reference)				1 (reference)			
Yes	0.855 (0.633–1.155)				0.948 (0.700–1.285)			
Menopause		0.0210		0.0037		0.1971		
No	1 (reference)		1 (reference)		1 (reference)			
Yes	1.531 (1.066–2.199)		1.412 (1.119–1.782)		1.274 (0.882–1.841)			
ALT (U/L)		0.9828				0.4137		
<15	1 (reference)				1 (reference)			
≥15	1.003 (0.740–1.361)				0.880 (0.648–1.196)			
AST (U/L)		0.3652				0.7735		
<18	1 (reference)				1 (reference)			
≥18	0.867 (0.636–1.181)				0.955 (0.696–1.309)			
LDH (U/L)		0.2055				0.3921		
<167	1 (reference)				1 (reference)			
≥167	1.198 (0.906–1.586)				1.131 (0.853–1.499)			
GGT (U/L)		0.8440				0.9701		
<17	1 (reference)				1 (reference)			
≥17	1.029 (0.773–1.370)				1.006 (0.751–1.347)			
ALP (U/L)		0.0780				0.0714		
<64	1 (reference)				1 (reference)			
≥64	1.293 (0.972–1.721)				1.306 (0.977–1.745)			
GLU (mmol/L)		0.0022		0.0032		0.0142		0.0019
<5.33	1 (reference)		1 (reference)		1 (reference)		1 (reference)	
≥5.33	0.647 (0.490–0.855)		0.713 (0.569–0.893)		0.694 (0.519–0.930)		0.683 (0.536–0.869)	
IgA		0.5811				0.3024		
<2.30	1 (reference)				1 (reference)			
≥2.30	1.074 (0.834–1.384)				1.146 (0.885–1.483)			
IgG		0.7248				0.7598		
<11.70	1 (reference)				1 (reference)			
≥11.70	0.956 (0.745–1.227)				0.962 (0.748–1.237)			
IgM		0.6205				0.7928		
<1.10	1 (reference)				1 (reference)			
≥1.10	0.939 (0.732–1.205)				0.966 (0.748–1.249)			
ALB		0.2803				0.7265		
<45.2	1 (reference)				1 (reference)			
≥45.2	1.172 (0.879–1.564)				0.949 (0.707–1.273)			
CRP		0.1714				0.4541		
<0.02	1 (reference)				1 (reference)			
≥0.02	0.822 (0.620–1.089)				0.894 (0.666–1.199)			
CA125		0.0174		0.0248		0.1988		
<13.35	1 (reference)		1 (reference)		1 (reference)			
≥13.35	1.372 (1.057–1.781)		1.298 (1.034–1.630)		1.188 (0.914–1.543)			
CA153		0.0040		0.0180		0.0042		0.0033
<11.63	1 (reference)		1 (reference)		1 (reference)		1 (reference)	
≥11.63	1.516 (1.143–2.012)		1.302 (1.046–1.620)		1.514 (1.140–2.011)		1.390 (1.116–1.732)	
CEA		0.4982				0.8598		
<1.66	1 (reference)				1 (reference)			
≥1.66	0.914 (0.705–1.186)				1.024 (0.786–1.334)			
D–D (mg/L)		0.1937				0.2868		
<0.29	1 (reference)				1 (reference)			
≥0.29	1.200 (0.911–1.581)				1.166 (0.879–1.546)			
FIB (g/L)		0.8146				0.2548		
<2.85	1 (reference)				1 (reference)			
≥2.85	0.969 (0.745–1.261)				1.167 (0.895–1.522)			
INR		0.6036				0.0448		0.0107
<0.93	1 (reference)				1 (reference)		1 (reference)	
≥0.93	0.936 (0.728–1.203)				1.296 (1.006–1.671)		1.335 (1.069–1.667)	
FDP (ug/ml)		0.5275				0.3305		
<1.40	1 (reference)				1 (reference)			
≥1.40	1.102 (0.815–1.492)				0.859 (0.633–1.166)			
ABO blood type		0.0874				0.1258		
A	1 (reference)				1 (reference)			
B	0.950 (0.695–1.299)				0.898 (0.649–1.243)			
O	0.718 (0.517–0.997)				0.745 (0.531–1.044)			
AB	1.175 (0.746–1.850)				1.238 (0.770–1.992)			
White blood cell (W)		0.0901				0.2279		
<6.01	1 (reference)				1 (reference)			
≥6.01	1.406 (0.948–2.086)				1.289 (0.853–1.947)			
Red blood cell (R)		0.8669				0.7343		
<4.40	1 (reference)				1 (reference)			
≥4.40	0.974 (0.716–1.325)				1.055 (0.774–1.438)			
Hemoglobin (Hb)		0.6310				0.3908		
<132	1 (reference)				1 (reference)			
≥132	0.928 (0.683–1.261)				0.877 (0.649–1.184)			
Neutrophil (N)		0.8081				0.8474		
<3.68	1 (reference)				1 (reference)			
≥3.68	0.956 (0.667–1.371)				0.964 (0.661–1.405)			
Lymphocyte (L)		0.1995				0.7082		
<1.76	1 (reference)				1 (reference)			
≥1.76	0.828 (0.620–1.105)				0.946 (0.707–1.265)			
Monocyte (M)		0.3330				0.0030		0.0030
<0.35	1 (reference)				1 (reference)		1 (reference)	
≥0.35	0.875 (0.668–1.146)				0.657 (0.497–0.868)		0.701 (0.556–0.884)	
Eosinophils (E)		0.0141		0.0197		0.0005		0.0234
<0.06	1 (reference)		1 (reference)		1 (reference)		1 (reference)	
≥0.06	0.715 (0.546–0.934)		0.766 (0.613–0.958)		0.613 (0.466–0.807)		0.775 (0.622–0.966)	
Basophils (B)		0.3230				0.2915		
<0.02	1 (reference)				1 (reference)			
≥0.02	1.156 (0.867–1.543)				1.172 (0.873–1.572)			
Platelet (P)		0.1400				0.2032		
<243	1 (reference)				1 (reference)			
≥243	0.829 (0.646–1.064)				0.847 (0.657–1.094)			
Nutritional risk index (NRI)		0.0191		0.0038		0.0257		0.0003
<112	1 (reference)		1 (reference)		1 (reference)		1 (reference)	
≥112	1.521 (1.071–2.161)		1.415 (1.119–1.789)		1.500 (1.051–2.143)		1.547 (1.221–1.959)	
Tumor site		0.1413				0.1316		
Right	1 (reference)				1 (reference)			
Left	1.208 (0.939–1.553)				1.218 (0.942-1.575)			
US-Primary tumor site		0.2583				0.2737		
Upper outer quadrant	1 (reference)				1 (reference)			
Lower outer quadrant	1.267 (0.852–1.885)				1.256 (0.832–1.895)			
Lower inner quadrant	1.399 (0.809–2.420)				1.747 (1.011–3.017)			
Upper inner quadrant	1.351 (0.964–1.891)				1.190 (0.841–1.686)			
Central	1.397 (0.798–2.447)				1.216 (0.692–2.137)			
US-Tumor size		0.5810				0.8227		
≤ 2 cm	1 (reference)				1 (reference)			
>2 and <5 cm	0.899 (0.657–1.228)				0.980 (0.713–1.346)			
≥5 cm	1.131 (0.616–2.077)				0.827 (0.445–1.537)			
US-LNM		0.9629				0.4328		
No	1 (reference)				1 (reference)			
Yes	0.992 (0.699–1.406)				1.152 (0.809–1.640)			
US-BIRADS		0.7120				0.5340		
4 (4a 4b 4c)	1 (reference)				1 (reference)			
5	0.828 (0.517–1.325)				0.766 (0.459–1.279)			
6	0.875 (0.540–1.419)				0.837 (0.494–11.419)			
Clinical stage								
Clinical T stage		0.0810				0.0403		0.0200
T1	1 (reference)				1 (reference)		1 (reference)	
T2	2.060 (1.190–3.568)				2.218 (1.241–3.964)		2.102 (1.181–3.740)	
T3	2.040 (1.026–4.055)				2.619 (1.285–5.341)		2.496 (1.227–5.079)	
T4	2.006 (0.901–4.464)				2.730 (1.177–6.332)		2.693 (1.167–6.212)	
Clinical *N* stage		0.1683				0.4248		
N0	1 (reference)				1 (reference)			
N1	0.957 (0.637–1.440)				1.051 (0.679–1.629)			
N2	0.976 (0.488–1.951)				0.998 (0.490–2.031)			
N3	1.676 (0.784–3.585)				1.552 (0.693–3.477)			
Clinical TNM stage		0.1995				0.3053		
I	1 (reference)				1 (reference)			
II	0.581 (0.310–1.091)				0.601 (0.308–1.172)			
III	0.693 (0.287–1.677)				0.662 (0.260–1.685)			
Operative time (min)		0.2776				0.0618		
<90	1 (reference)				1 (reference)			
≥90	0.855 (0.645–1.134)				0.760 (0.569–1.014)			
Type of surgery		0.1932				0.4770		
Mastectomy	1 (reference)				1 (reference)			
Breast-conserving surgery	0.788 (0.550–1.128)				1.144 (0.790–1.656)			
Histologic type		0.0200		0.0190		0.0083		0.0060
Ductal	1 (reference)		1 (reference)		1 (reference)		1 (reference)	
Lobular	2.682 (1.175–6.119)		2.718 (1.187–6.223)		2.638 (1.099–6.334)		2.562 (1.229–5.341)	
Others	2.230 (1.067–4.660)		2.074 (1.005–4.284)		2.552 (1.149–5.672)		2.162 (1.050–4.448)	
Histologic grade		0.1184				0.1867		
I	1 (reference)				1 (reference)			
II	0.784 (0.490–1.255)				0.811 (0.502–1.310)			
III	0.625 (0.379–1.030)				0.655 (0.391–1.097)			
Pathological T stage		0.0100		0.0099		0.0184		0.0380
Tis/T0	1 (reference)		1 (reference)		1 (reference)		1 (reference)	
T1	1.573 (0.897–2.758)		1.573 (0.897–2.758)		0.625 (0.204–1.916)		0.605 (0.197–1.854)	
T2	1.981 (1.126–3.486)		1.981 (1.126–3.486)		0.512 (0.161–1.629)		0.498 (0.158–1.572)	
T3	1.485 (0.732–3.014)		1.485 (0.732–3.014)		0.420 (0.117–1.505)		0.397 (0.111–1.426)	
T4	3.324 (1.557–7.096)		3.324 (1.557–7.096)		1.537 (0.392–6.027)		1.320 (0.334–5.221)	
Pathological *N* stage		0.0103		0.0140		<0.0001		<0.0001
N0	1 (reference)		1 (reference)		1 (reference)		1 (reference)	
N1	2.592 (0.865–7.767)		2.550 (0.841–7.734)		1.818 (0.619–5.344)		1.400 (1.047–1.872)	
N2	3.603 (0.923–14.063)		3.726 (0.947–14.660)		4.966 (1.444–17.085)		1.685 (1.192–2.381)	
N3	5.998 (1.535–23.435)		6.016 (1.527–23.694)		9.131 (2.615–31.877)		2.384 (1.717–3.311)	
Pathological TNM stage		0.0030		0.0170		0.0110		0.0005
Tis/T0	1 (reference)		1 (reference)		1 (reference)		1 (reference)	
I	1.998 (0.584–6.839)		1.322 (0.658–2.655)		2.671 (0.738–9.663)		2.849 (0.786–10.320)	
II	2.282 (0.634–8.210)		1.558 (0.778–3.121)		3.727 (0.969–14.331)		3.963 (1.044–15.046)	
III	2.025 (0.420–9.760)		0.631 (0.261–1.526)		1.258 (0.274–5.771)		1.215 (0.265–5.575)	
Total lymph nodes		0.8118				0.6789		
<21	1 (reference)				1 (reference)			
≥21	0.935 (0.536–1.629)				0.882 (0.487–1.598)			
Positive lymph nodes		0.3806				0.6448		
<1	1 (reference)				1 (reference)			
≥1	0.564 (0.157–2.028)				0.742 (0.209–2.638)			
Total axillary lymph nodes		0.2165				0.3777		
<20	1 (reference)				1 (reference)			
≥20	0.704 (0.404–1.228)				0.767 (0.425–1.383)			
Positive axillary lymph nodes		0.6622				0.6196		
<1	1 (reference)				1 (reference)			
≥1	0.822 (0.342–1.978)				0.788 (0.307–2.020)			
Molecular subtype		0.0520				0.0581		
Luminal A	1 (reference)				1 (reference)			
Luminal B HER2+	0.264 (0.097–0.720)				0.226 (0.080–0.638)			
Luminal B HER2–	0.630 (0.366–1.082)				0.514 (0.296–0.893)			
HER2 enriched	0.187 (0.063–0.558)				0.247 (0.081–0.753)			
Triple negative	0.581 (0.286–1.177)				0.547 (0.266–1.124)			
ER status		0.2301				0.9455		
Negative	1 (reference)				1 (reference)			
Positive	0.735 (0.444–1.215)				1.018 (0.616–1.680)			
PR status		0.2885				0.2090		
Negative	1 (reference)				1 (reference)			
Positive	1.237 (0.835–1.833)				1.269 (0.875–1.839)			
HER2 status		0.1047				0.1166		
Negative (0—-++)	1 (reference)				1 (reference)			
Positive (+++)	2.109 (0.856–5.196)				2.041 (0.837–4.975)			
Ki-67 status		0.0020		0.0370		0.0041		0.0380
Negative (≤ 14%)	1 (reference)		1 (reference)		1 (reference)		1 (reference)	
Positive (>14%)	1.731 (1.223–2.450)		1.332 (1.018–1.742)		1.664 (1.175–2.357)		1.329 (1.016–1.738)	
AR status		0.4306				0.9714		
Negative	1 (reference)				1 (reference)			
Positive	0.835 (0.534–1.307)				0.991 (0.607–1.618)			
CK5/6 status		0.0170		0.0007		0.0238		0.0002
Negative	1 (reference)		1 (reference)		1 (reference)		1 (reference)	
Positive	1.725 (1.103–2.699)		1.756 (1.271–2.428)		1.713 (1.074–2.732)		1.870 (1.349–2.593)	
E-cad status		0.1380				<0.0001		<0.0001
Negative	1 (reference)				1 (reference)		1 (reference)	
Positive	1.297 (0.920–1.830)				2.566 (1.765–3.728)		2.667 (2.002–3.553)	
EGFR status		0.2977				0.9685		
Negative	1 (reference)				1 (reference)			
Positive	0.805 (0.535–1.211)				1.009 (0.655–1.554)			
P53 status		0.0840				0.0729		
Negative	1 (reference)				1 (reference)			
Positive	0.783 (0.593–1.033)				0.774 (0.585–1.024)			
TOP2A status		0.4136				0.3998		
Negative	1 (reference)				1 (reference)			
Positive	1.159 (0.814–1.651)				1.173 (0.809–1.700)			
Lymph vessel invasion		0.0329		0.0002		0.0321		0.0011
Negative	1 (reference)		1 (reference)		1 (reference)		1 (reference)	
Positive	1.423 (1.029–1.966)		1.585 (1.245–2.018)		1.429 (1.031–1.981)		1.523 (1.182–1.962)	
Neural invasion		0.7620				0.5040		
Negative	1 (reference)				1 (reference)			
Positive	0.937 (0.613–1.432)				1.152 (0.761–1.742)			
Post-operative chemotherapy		<0.0001		0.0001		0.0001		0.0006
No	1 (reference)		1 (reference)		1 (reference)		1 (reference)	
Yes	0.458 (0.314–0.670)		0.523 (0.376–0.725)		0.475 (0.324–0.697)		0.575 (0.420–0.789)	
Post-operative radiotherapy		0.2115				0.1298		
No	1 (reference)				1 (reference)			
Yes	1.236 (0.886–1.723)				1.303 (0.925–1.834)			
Post-operative endocrine therapy		0.0105		0.0300		0.0210		0.0280
No	1 (reference)		1 (reference)		1 (reference)		1 (reference)	
Yes	0.631 (0.444–0.898)		0.771 (0.609–0.975)		0.752 (0.590–0.958)		0.764 (0.602–0.971)	
Post-operative targeted therapy		<0.0001		<0.0001		<0.0001		<0.0001
No	1 (reference)		1 (reference)		1 (reference)		1 (reference)	
Yes	0.507 (0.390–0.658)		0.457 (0.356–0.587)		0.590 (0.457–0.763)		0.556 (0.432-0.716)	

### DFS and OS by NRI

As seen in Table 3, the NRI was the important prognostic factors DFS and OS using the cutoff value of 112. The results performed that high NRI was associated with prolonged DFS and OS (*P* = 0.019, HR: 1.521, 95% CI: 1.071–2.161 and *P* = 0.004, HR: 1.415, 95% CI: 1.119–1.789; and *P* = 0.026, HR: 1.500, 95% CI: 1.051–2.143 and *P* < 0.001, HR: 1.547, 95% CI: 1.221–1.959, respectively), on both univariate and multivariate analyses.

**Figure 1:**
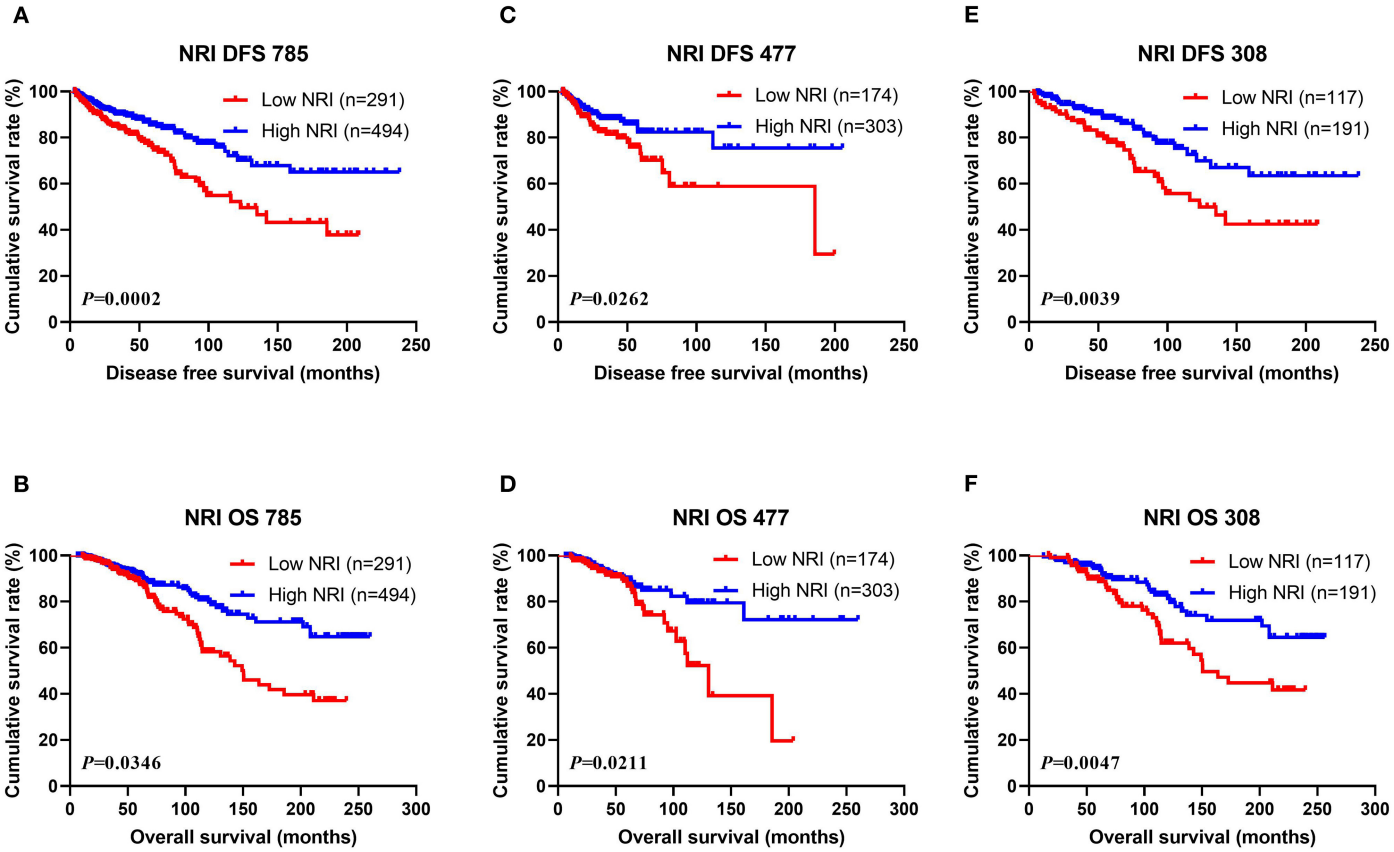
DFS and OS of patients with breast cancer. **(A)** Kaplan-Meier analysis of DFS for the NRI of all breast cancer patients. **(B)** Kaplan-Meier analysis of OS for the NRI of all breast cancer patients. **(C)** Kaplan-Meier analysis of DFS for the NRI of breast cancer patients in NACT group. **(D)** Kaplan-Meier analysis of OS for the NRI of breast cancer patients in NACT group. **(E)** Kaplan-Meier analysis of DFS for the NRI of breast cancer patients in non-NACT group. **(F)** Kaplan-Meier analysis of OS for the NRI of breast cancer patients in non-NACT group.

Of all breast cancer patients, patients with low NRI scores had mean DFS and OS time of 40.50 and 63.47 months, while patients with high NRI scores were 56.39 and 71.50 months, respectively. Furthermore, the mean DFS and OS survive time of NRI in the high group were remarkably longer in contrast to for those of NRI in the low group by the log-rank analysis (χ^2^ = 13.9500, *P* = 0.0002 and χ^2^ = 4.4660, *P* = 0.0346, respectively; Figures 1A,B). In the NACT group, the mean DFS and OS survive time of NRI in the high group were remarkably longer in contrast to those of NRI in the low group (χ^2^ = 4.9440, *P* = 0.0262 and χ^2^ = 5.3210, *P* = 0.0211, respectively; Figures 1C,D). In the non-NACT group, the mean DFS and OS survive time of NRI in the high group were remarkably longer in contrast to those of NRI in the low group (χ^2^ = 8.3230, *P* = 0.0039 and χ^2^ = 7.9940, *P* = 0.0047, respectively; Figures 1E,F).

### The Association Between Pathologic Stage and NRI in Breast Cancer Patients

**Figure 2:**
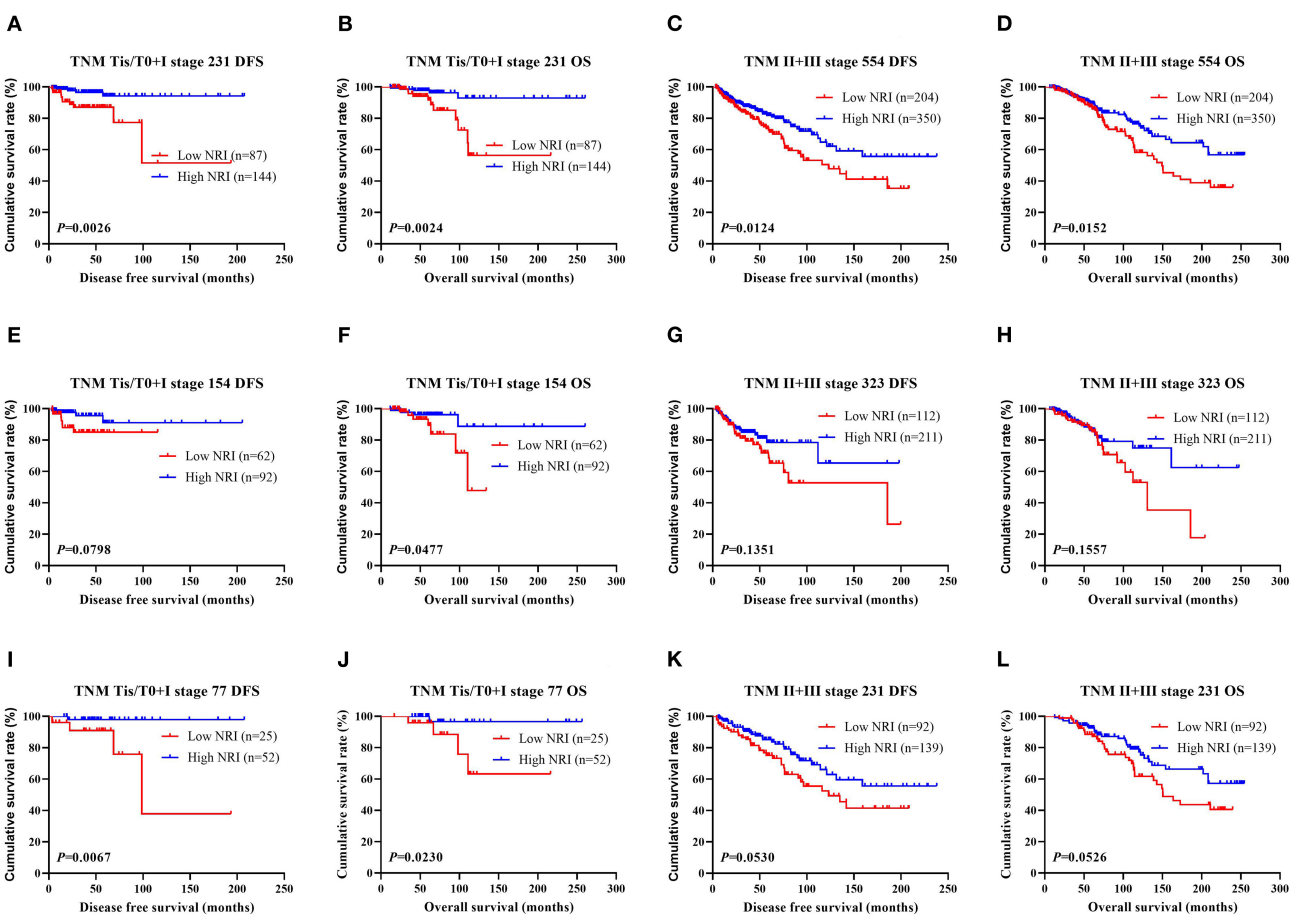
DFS and OS for the NRI of breast cancer patients in different pathologic stages. **(A)** Kaplan-Meier analysis of DFS for the NRI scores of early-stage breast cancer (Tis/T0+I stage) patients in all enrolled breast cancer patients. **(B)** Kaplan-Meier analysis of OS for the NRI values of early-stage breast cancer (Tis/T0+I stage) patients in all enrolled breast cancer patients. **(C)** Kaplan-Meier analysis of DFS for the NRI values of advanced stage breast cancer (II+III stage) patients in all enrolled breast cancer patients. **(D)** Kaplan-Meier analysis of OS for the NRI levels of advanced stage breast cancer (II + III stage) patients in all enrolled breast cancer patients. **(E)** Kaplan-Meier analysis of DFS for the NRI values of early-stage breast cancer (Tis/T0 + I stage) patients in NACT group. **(F)** Kaplan-Meier analysis of OS for the NRI scores of early-stage breast cancer (Tis/T0 + I stage) patients in NACT group. **(G)** Kaplan-Meier analysis of DFS for the NRI values of advanced stage breast cancer (II + III stage) patients in NACT group. **(H)** Kaplan-Meier analysis of OS for the NRI values of advanced stage breast cancer (II + III stage) patients in NACT group. **(I)** Kaplan-Meier analysis of DFS for the NRI scores of early-stage breast cancer (Tis/T0 + I stage) patients in non-NACT group. **(J)** Kaplan-Meier analysis of OS for the NRI scores of early-stage breast cancer (Tis/T0 + I stage) patients in non-NACT group. **(K)** Kaplan-Meier analysis of DFS for the NRI values of advanced stage breast cancer (II + III stage) patients in non-NACT group. **(L)** Kaplan-Meier analysis of OS for the NRI of advanced stage breast cancer (II + III stage) patients in non-NACT group.

The results shown that pathologic TNM stage was the significant predictor via the univariate and multivariate analyses (see Table 3). In order to further study the efficiency of prediction of NRI, and the NRI was analyzed by the pathologic TNM stage. Of all breast cancer patients, the results shown that patients with high NRI scores had notably longer DFS and OS survive time than those with low NRI scores in early-stage breast cancer (included pathologic Tis/T0 and pathologic I stage) (χ^2^ = 9.0510, *P* = 0.0026 and χ^2^ = 9.2140, *P* = 0.0024). Similarly, patients with high NRI scores had remarkably longer DFS and OS survive time than those with low NRI scores in advanced stage breast cancer (pathologic II and pathologic III stage) (χ^2^ = 6.2500, *P* = 0.0124 and χ^2^ = 5.8880, *P* = 0.0152). In the NACT group, the results also indicated that patients with high NRI scores had longer DFS and OS survive time than those with low NRI scores in early-stage breast cancer (χ^2^ = 3.0700, *P* = 0.0798 and χ^2^ = 3.9210, *P* = 0.0477). Meanwhile, patients with high NRI scores had longer DFS and OS survive time than those with low NRI scores in advanced stage breast cancer (χ^2^ = 2.2330, *P* = 0.1351 and χ^2^ = 2.0160, *P* = 0.1557). In the non-NACT group, the results demonstrated that patients with high NRI scores had remarkably longer DFS and OS survive time than those with low NRI scores in early-stage breast cancer (χ^2^ = 7.3580, *P* = 0.0067 and χ^2^ = 5.1700, *P* = 0.0230). Furthermore, patients with high NRI scores had longer DFS and OS than those with low NRI scores in advanced stage breast cancer (χ^2^ = 3.7450, *P* = 0.0530 and χ^2^ = 3.7570, *P* = 0.0526). See in Figure 2.

### The Association Between Pathology Parameters and NRI in Patients With Breast Cancer

**Figure 3:**
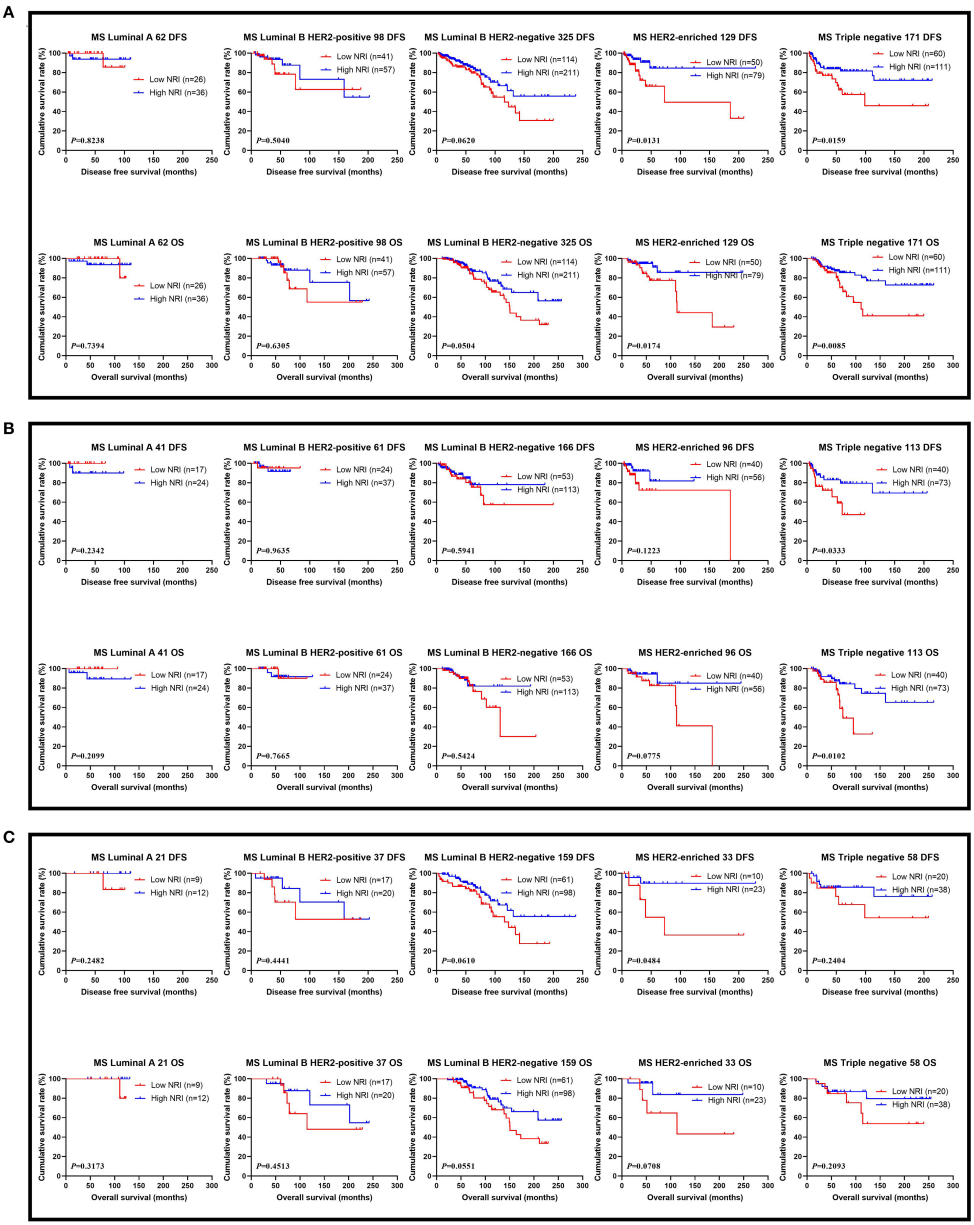
DFS and OS for the NRI of breast cancer patients in different molecular subtypes. **(A)** DFS and OS for the NRI of breast cancer patients in different molecular subtypes in all patients; **(B)** DFS and OS for the NRI of breast cancer patients in different molecular subtypes in NACT group; **(C)** DFS and OS for the NRI of breast cancer patients in different molecular subtypes in non-NACT group.

The results performed that statistically significant differences were found in TOP2A status (χ^2^ = 4.0108, *P* = 0.0452), and no statistically significant differences were observed in the other pathology parameters in all cases (*P* > 0.05). These findings were shown in Table 4. We also analyzed that the different molecular subtypes by NRI. Of all enrolled patients, the mean DFS and OS survive time for patients with high NRI by the log-rank test were longer than in those with low NRI in Luminal A subtype (χ^2^ = 0.0496, *P* = 0.8238 and χ^2^ = 0.1107, *P* = 0.7394), Luminal B HER2-positive subtype (χ^2^ = 0.4465, *P* = 0.5040 and χ^2^ = 0.2313, *P* = 0.6305), Luminal B HER2-negative subtype (χ^2^ = 3.4830, *P* = 0.0620 and χ^2^ = 3.8280, *P* = 0.0504), HER2-enriched subtype (χ^2^ = 6.1510, *P* = 0.0131 and χ^2^ = 5.6560, *P* = 0.0174), triple-negative subtype (χ^2^ = 5.8120, *P* = 0.0159 and χ^2^ = 6.9300, *P* = 0.0085; Figure 3A). Moreover, we also analyzed the molecular subtypes by NRI in the NACT group and the non-NACT group (Figures 3B,C).

**Table 4:** The association between molecular subtype and NRI in patients with breast cancer.

**Parameters**	**NRI 785**				**NRI 477**				**NRI 308**			
**Cases (*n*)**	**Low NRI 291**	**High NRI 494**	**χ^2^**	***P*-value**	**Low NRI 174**	**High NRI 303**	**χ^2^**	***P*-value**	**Low NRI 117**	**High NRI 191**	**χ^2^**	***P*-value**
Core needle biopsy (*N* = 477)												
Molecular subtype							4.0360	0.4012				
Luminal A					12 (6.90%)	13 (4.29%)						
Luminal B HER2+					23 (13.22%)	44 (14.52%)						
Luminal B HER2–					62 (35.63%)	124 (40.92%)						
HER2 enriched					39 (22.41%)	52 (17.16%)						
Triple negative					38 (21.84%)	70 (23.10%)						
ER status							0.2041	0.6515				
Negative					72 (41.38%)	119 (39.27%)						
Positive					102 (58.62%)	184 (60.73%)						
PR status							0.0337	0.8544				
Negative					68 (39.08%)	121 (39.93%)						
Positive					106 (60.92%)	182 (60.07%)						
HER2 status							0.6994	0.4030				
Negative (0—++)					110 (63.22%)	203 (67.00%)						
Positive (+++)					64 (36.78%)	100 (33.00%)						
Ki-67 status							0.3469	0.5559				
Negative (≤ 14%)					33 (18.97%)	51 (16.83%)						
Positive (>14%)					141 (81.03%)	252 (83.17%)						
Postoperative pathology (IHC)												
Molecular subtype			2.9300	0.5696			5.1830	0.2690			2.9020	0.5743
Luminal A	26 (8.93%)	36 (7.29%)			17 (9.77%)	24 (7.92%)			9 (7.69%)	12 (6.28%)		
Luminal B HER2+	41 (14.09%)	57 (11.54%)			24 (13.79%)	37 (12.21%)			17 (14.53%)	20 (10.47%)		
Luminal B HER2–	111 (38.14%)	214 (43.32%)			50 (28.74%)	116 (38.28%)			61 (52.14%)	98 (51.31%)		
HER2 enriched	50 (17.18%)	79 (15.99%)			41 (23.56%)	55 (18.15%)			9 (7.69%)	24 (12.57%)		
Triple negative	63 (21.65%)	108 (21.86%)			42 (24.14%)	71 (23.43%)			21 (17.95%)	37 (19.37%)		
ER status			0.1729	0.6775			0.8871	0.3463			3.3940	0.0654
Negative	107 (36.77%)	189 (38.26%)			76 (43.68%)	119 (39.27%)			31 (26.50%)	70 (36.65%)		
Positive	184 (63.23%)	305 (61.74%)			98 (56.32%)	184 (60.73%)			86 (73.50%)	121 (63.35%)		
PR status			0.7569	0.3843			0.0058	0.9395			2.1254	0.1449
Negative	111 (38.14%)	204 (41.30%)			77 (44.25%)	133 (43.89%)			34 (29.06%)	71 (37.17%)		
Positive	180 (61.86%)	290 (58.70%)			97 (55.75%)	170 (56.11%)			83 (70.94%)	120 (62.83%)		
HER2 status			0.7958	0.3724			1.3451	0.2461			0.0172	0.8956
Negative (0—++)	201 (69.07%)	356 (72.06%)			111 (63.79%)	209 (68.98%)			90 (76.92%)	147 (76.96%)		
Positive (+++)	90 (30.93%)	138 (27.94%)			63 (36.21%)	94 (31.02%)			27 (23.08%)	44 (23.04%)		
Ki-67 status			3.7906	0.0515			2.7846	0.0952			1.2634	0.2610
Negative (≤ 14%)	93 (31.96%)	126 (25.51%)			64 (36.78%)	89 (29.37%)			29 (24.79%)	37 (19.37%)		
Positive (>14%)	198 (68.04%)	368 (74.49%)			110 (63.22%)	214 (70.63%)			88 (75.21%)	154 (80.63%)		
AR status			2.1484	0.1427			1.7504	0.1858			0.2902	0.5901
Negative	254 (87.29%)	412 (83.40%)			138 (79.31%)	224 (73.93%)			116 (99.15%)	188 (98.43%)		
Positive	37 (12.71%)	82 (16.60%)			36 (20.69%)	79 (26.07%)			1 (0.85%)	3 (1.57%)		
CK5/6 status			0.2902	0.5901			0.0007	0.9786			0.9001	0.3428
Negative	256 (87.97%)	428 (86.64%)			148 (85.06%)	258 (85.15%)			108 (92.31%)	170 (89.01%)		
Positive	35 (12.03%)	66 (13.36%)			26 (14.94%)	45 (14.85%)			9 (7.69%)	21 (10.99%)		
E-cad status			0.0005	0.9831			0.1598	0.6894			0.1258	0.7228
Negative	131 (45.02%)	222 (44.94%)			60 (34.48%)	110 (36.30%)			71 (60.68%)	112 (58.64%)		
Positive	160 (54.98%)	272 (55.06%)			114 (65.52%)	193 (63.70%)			46 (39.32%)	79 (41.36%)		
EGFR status			2.1847	0.1394			0.9965	0.3182			1.1764	0.2781
Negative	227 (78.01%)	362 (73.28%)			127 (72.99%)	208 (68.65%)			100 (85.47%)	154 (80.63%)		
Positive	64 (21.99%)	132 (26.72%)			47 (27.01%)	95 (31.35%)			17 (14.53%)	37 (19.37%)		
P53 status			0.2789	0.5974			0.0668	0.7960			0.2816	0.5957
Negative	150 (51.55%)	245 (49.60%)			90 (51.72%)	153 (50.50%)			60 (51.28%)	92 (48.17%)		
Positive	141 (48.45%)	249 (50.40%)			84 (48.28%)	150 (49.50%)			57 (48.72%)	99 (51.83%)		
TOP2A status			4.0108	0.0452			0.0014	0.9699			9.6194	0.0019
Negative	124 (42.61%)	175 (35.43%)			60 (34.48%)	105 (34.65%)			64 (54.70%)	70 (36.65%)		
Positive	167 (57.39%)	319 (64.57%)			114 (65.52%)	198 (65.35%)			53 (45.30%)	121 (63.35%)		
Lymph vessel invasion			0.3940	0.5302			0.1226	0.7263			0.4555	0.4998
Negative	203 (69.76%)	355 (71.86%)			115 (66.09%)	205 (67.66%)			88 (75.21%)	150 (78.53%)		
Positive	88 (30.24%)	139 (28.14%)			59 (33.91%)	98 (32.34%)			29 (24.79%)	41 (21.47%)		
Neural invasion			1.2591	0.2618			0.2483	0.6183			2.7576	0.0968
Negative	243 (83.51%)	427 (86.44%)			138 (79.31%)	246 (81.19%)			105 (89.74%)	181 (94.76%)		
Positive	48 (16.49%)	67 (13.56%)			36 (20.69%)	57 (18.81%)			12 (10.26%)	10 (5.24%)		

### The Association Between LVI and NRI in Breast Cancer Patients

**Figure 4:**
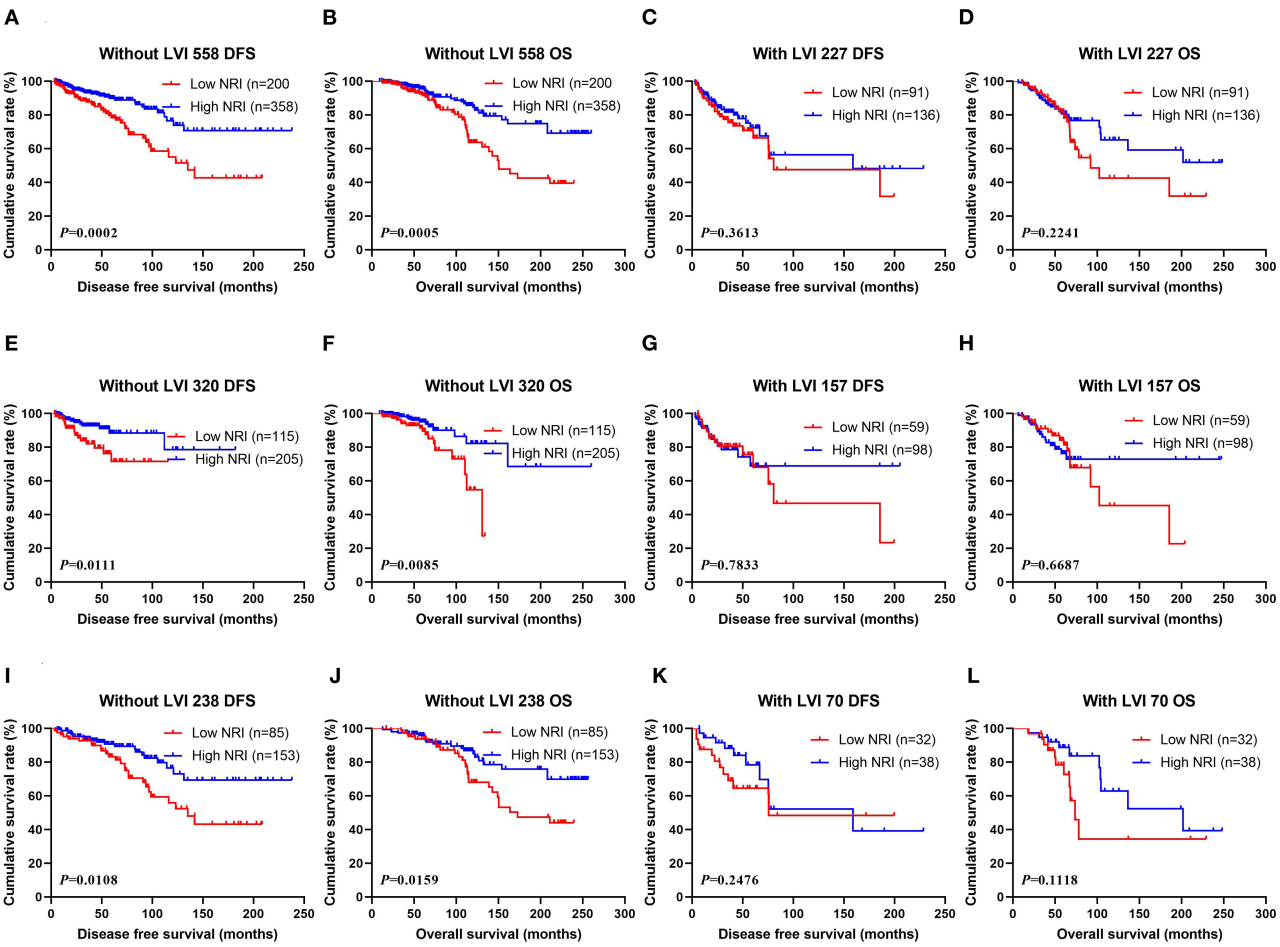
**(A)** Kaplan-Meier analysis of DFS of patients without LVI by NRI in all breast cancer patients, **(B)** Kaplan-Meier analysis of OS of patients without LVI by NRI in all breast cancer patients, **(C)** Kaplan-Meier analysis of DFS of patients with LVI by NRI in all breast cancer patients, **(D)** Kaplan-Meier analysis of OS of patients LVI by NRI in all breast cancer patients; **(E)** Kaplan-Meier analysis of DFS of patients without LVI by NRI in NACT group, **(F)** Kaplan-Meier analysis of OS of patients without LVI by NRI in NACT group, **(G)** Kaplan-Meier analysis of DFS of patients with LVI by NRI in NACT group, **(H)** Kaplan-Meier analysis of OS of patients with LVI by NRI in NACT group; **(I)** Kaplan-Meier analysis of DFS of patients without LVI by NRI in non-NACT group, **(J)** Kaplan-Meier analysis of OS of patients without LVI by NRI in non-NACT group, **(K)** Kaplan-Meier analysis of DFS of patients with LVI by NRI in non-NACT group, **(L)** Kaplan-Meier analysis of OS of patients with LVI by NRI in non-NACT group.

Through univariate and multivariate analyses, LVI was the significant predictor (Table 3). The ability of NRI to determine breast cancer prognosis was further assessed by examining the relationship between LVI and NRI. Among the patients without LVI, patients who had high NRI scores had remarkably longer DFS and OS survive time than those had low NRI scores (χ^2^ = 13.6600, *P* = 0.0002 and χ^2^ = 12.1500, *P* = 0.0005). Among the patients with LVI, patients who had high NRI scores had longer DFS and OS survive time than those had low NRI scores (χ^2^ = 0.8332, *P* = 0.3613 and χ^2^ = 1.4780, *P* = 0.2241). In the NACT group, patients who had high NRI scores had notably longer DFS and OS survive time than those had low NRI scores without LVI (χ^2^ = 6.4450, *P* = 0.0111 and χ^2^ = 6.9200, *P* = 0.0085). Furthermore, patients who had high NRI scores had longer DFS and OS survive time than those had low NRI scores with LVI (χ^2^ = 0.07560, *P* = 0.7833 and χ^2^ = 0.1831, *P* = 0.6687). In the non-NACT group, patients who had high NRI values had remarkably longer DFS and OS survive time than those had low NRI values without LVI (χ^2^ = 6.4910, *P* = 0.0108 and χ^2^ = 5.8110, *P* = 0.0159). At the same time, patients who had high NRI values had longer DFS and OS survive time than those had low NRI values with LVI (χ^2^ = 1.3370, *P* = 0.2476 and χ^2^ = 2.5280, *P* = 0.1118; Figure 4).

### The Association Between NRI and Response in Breast Cancer Patients Received NACT

**Figure 5:**
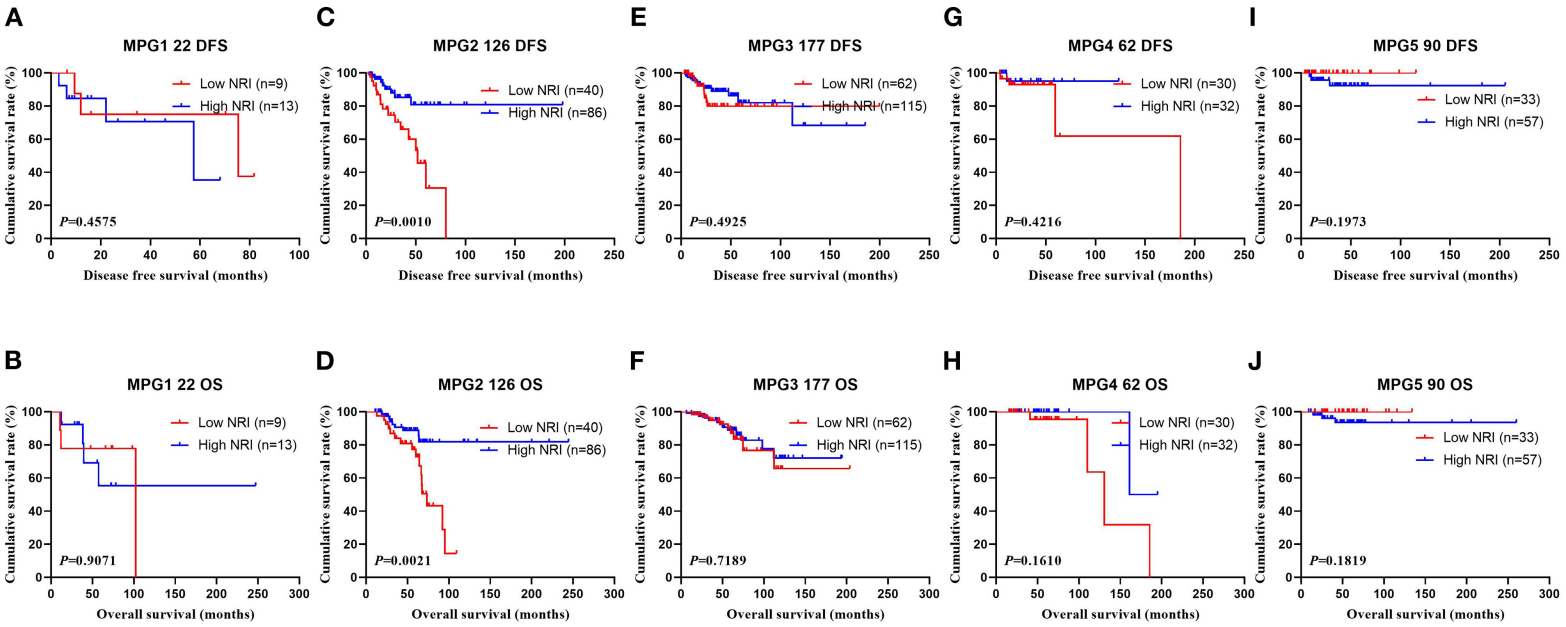
DFS and OS for the NRI of breast cancer patients in Miller and Payne grade (MPG) in NACT group. **(A)** Kaplan-Meier analysis of DFS of patients with MPG 1, **(B)** Kaplan-Meier analysis of OS of patients with MPG 1, **(C)** Kaplan-Meier analysis of DFS of patients with MPG 2, **(D)** Kaplan-Meier analysis of OS of patients with MPG 2, **(E)** Kaplan-Meier analysis of DFS of patients with MPG 3, **(F)** Kaplan-Meier analysis of OS of patients with MPG 3, **(G)** Kaplan-Meier analysis of DFS of patients with MPG 4, **(H)** Kaplan-Meier analysis of OS of patients with MPG 4, **(I)** Kaplan-Meier analysis of DFS of patients with MPG 5, **(J)** Kaplan-Meier analysis of OS of patients with MPG 5.

**Figure 6:**
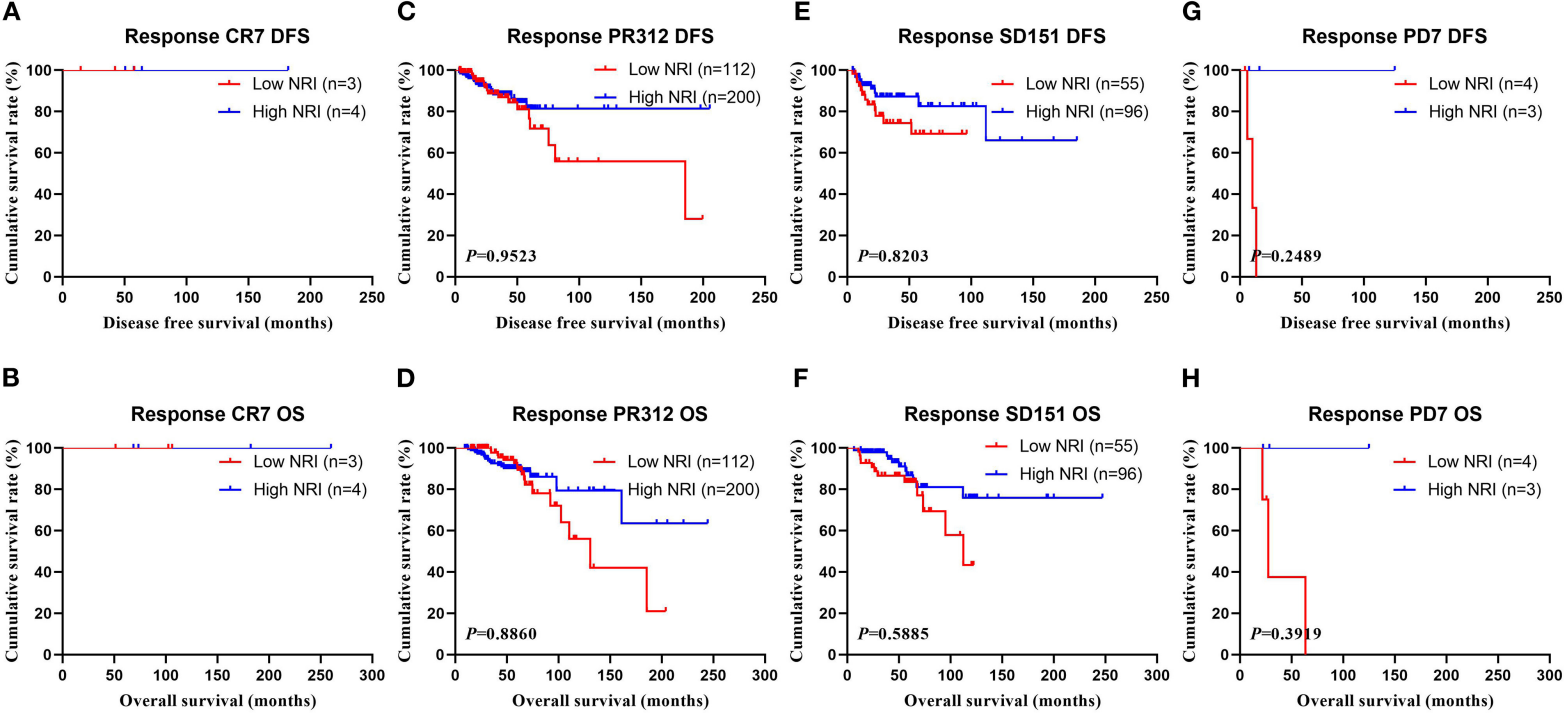
DFS and OS for the NRI of breast cancer patients in response in NACT group. **(A)** Kaplan-Meier analysis of DFS of patients with response CR, **(B)** Kaplan-Meier analysis of OS of patients with response CR, **(C)** Kaplan-Meier analysis of DFS of patients with response PR, **(D)** Kaplan-Meier analysis of OS of patients with response PR, **(E)** Kaplan-Meier analysis of DFS of patients with response SD, **(F)** Kaplan-Meier analysis of OS of patients with response SD, **(G)** Kaplan-Meier analysis of DFS of patients with response PD, **(H)** Kaplan-Meier analysis of OS of patients with response PD.

In the NACT group, all enrolled received neoadjuvant chemotherapy, and the effect of chemotherapy was evaluated after two chemotherapy cycles. After surgery, the degree of pathological remission was evaluated by MPG. So, we analyzed the MPG by NRI, and the results indicated that there was no difference in MPG grade 1 (χ^2^ = 0.5520, *P* = 0.4575 and χ^2^ = 0.0136, *P* = 0.9071), MPG grade 3 (χ^2^ = 0.4711, *P* = 0.4925 and χ^2^ = 0.1296, *P* = 0.7189), MPG grade 4 (χ^2^ = 0.6459, *P* = 0.4216 and χ^2^ = 1.9650, *P* = 0.1610), MPG grade 5 (χ^2^ = 1.6620, *P* = 0.1973 and χ^2^ = 1.7820, *P* = 0.1819), except in MPG grade 2 (χ^2^ = 10.9100, *P* = 0.0010 and χ^2^ = 9.5030, *P* = 0.0021; Figure 5). Furthermore, we analyzed the relationship between response and NRI, and the results indicated that there was no difference in CR (χ^2^ = 0.0000, P>0.9999 and χ^2^ = 0.0000, P>0.9999), PR (χ^2^ = 0.7815, *P* = 0.3767 and χ^2^ = 0.6523, *P* = 0.4193), SD (χ^2^ = 2.5450, *P* = 0.1107 and χ^2^ = 3.1730, *P* = 0.0749), except in PD (χ^2^ = 3.8460, *P* = 0.0499 and χ^2^ = 2.7400, *P* = 0.0979; Figure 6).

### The Relationship Between NRI and Toxicity and Adverse Effects

In the NACT group, the common toxicities after NACT were hematologic and gastrointestinal reactions. The results shown that the nausea (χ^2^ = 9.2413, *P* = 0.0024), mouth ulcers (χ^2^ = 4.8133, *P* = 0.0282), anemia (χ^2^ = 8.5441, *P* = 0.0140), and leukopenia (χ^2^ = 11.0951, *P* = 0.0039) were statistically different between the two groups (see Table 5).

**Table 5:** The correlation between NRI and toxicity assessment.

**Parameters**	**NRI 477**			
**Cases (*n*)**	**Low NRI 174**	**High NRI 303**	**χ^2^**	***P*-value**
Decreased appetite			2.2133	0.1368
No	20 (11.49%)	50 (16.50%)		
Yes	154 (88.51%)	253 (83.50%)		
Nausea			9.2413	0.0024
No	11 (6.32%)	48 (15.84%)		
Yes	163 (93.68%)	255 (84.16%)		
Vomiting			2.5293	0.1118
No	77 (44.25%)	157 (51.82%)		
Yes	97 (55.75%)	146 (48.18%)		
Diarrhea			0.5410	0.4620
No	160 (91.95%)	284 (93.73%)		
Yes	14 (8.05%)	19 (6.27%)		
Mouth ulcers			4.8133	0.0282
No	165 (94.83%)	298 (98.35%)		
Yes	9 (5.17%)	5 (1.65%)		
Alopecia			0.0350	0.8516
No	80 (45.98%)	142 (46.86%)		
Yes	94 (54.02%)	161 (53.14%)		
Peripheral neurotoxicity			0.1828	0.6690
No	144 (82.76%)	246 (81.19%)		
Yes	30 (17.24%)	57 (18.81%)		
Anemia			8.5441	0.0140
Grade 0	79 (45.40%)	178 (58.75%)		
Grade 1–2	92 (52.87%)	123 (40.59%)		
Grade 3–4	3 (1.72%)	2 (0.66%)		
Leukopenia			11.0951	0.0039
Grade 0	35 (20.11%)	103 (33.99%)		
Grade 1–2	92 (52.87%)	141 (46.53%)		
Grade 3–4	47 (27.01%)	59 (19.47%)		
Neutropenia			5.3754	0.0680
Grade 0	41 (23.56%)	102 (33.66%)		
Grade 1–2	71 (40.80%)	108 (35.64%)		
Grade 3–4	62 (35.63%)	93 (30.69%)		
Thrombocytopenia			3.8748	0.1441
Grade 0	128 (73.56%)	244 (80.53%)		
Grade 1–2	44 (25.29%)	54 (17.82%)		
Grade 3–4	2 (1.15%)	5 (1.65%)		
Gastrointestinal reaction			4.2926	0.1169
Grade 0	8 (4.60%)	30 (9.90%)		
Grade 1–2	164 (94.25%)	269 (88.78%)		
Grade 3–4	2 (1.15%)	4 (1.32%)		
Myelosuppression			2.2843	0.3191
Grade 0	27 (15.52%)	63 (20.79%)		
Grade 1–2	64 (36.78%)	111 (36.63%)		
Grade 3–4	83 (47.70%)	129 (42.57%)		
Hepatic dysfunction			2.8849	0.2364
Grade 0	129 (74.14%)	242 (79.87%)		
Grade 1–2	45 (25.86%)	60 (19.80%)		
Grade 3–4	0 (0.00%)	1 (0.33%)		

## Discussion

Breast cancer is a major public health threat globally (29). In women around the world, breast cancer is a very common female malignant tumor and the leading cause of cancer-related deaths (2). Although promising treatment options are emerging, recurrence and metastasis are still the driving causes for breast cancer fatality (30). Evidence shows that approximately 30%-40% of patients who suffer from invasive breast cancer will eventually progress to metastatic breast cancer, whose 5-year survival rate could be poorer than 30% (31, 32). Additionally, research also suggests that probabilities of recurrence and progression could occur in some breast cancer patients even after radical resection and neoadjuvant/adjuvant therapy (33). Therefore, to address these issues, there is an urgent need to develop assessment strategies based on non-invasive, reproducible, and convenient biomarkers to estimate the curative effects and the prognosis of breast cancer, as well as to better pair treatment options with patient characteristics (e.g., ascertain those breast cancer patients who get a profit from neoadjuvant chemotherapy).

Prior studies have identified a limited number of screening tools to evaluate nutritional risks that have the potential to predict prognosis in cancer patients, ranging from Subjective Global Assessment (SGA), Nutritional Risk Screening 2002 (NRS 2002), Mini Nutritional Assessment-Screening Form (MNA-SF), and Malnutrition Universal Screening Tool (MUST), as well as several nutritional status markers such as the neutrophil-to-lymphocyte ratio, prognostic nutritional index, BMI, serum albumin, total lymphocyte count, and indicators such as patients' cholesterol levels (34–38). Among them, BMI and serum albumin level are usually used as makers of patients' nutritional status in routine clinical practice (39), largely due to their abilities to predict cancer patients' survival rates, as indicated in recent studies (40–42). While these tools play an important role in nutritional assessment, the fact that they rely on subjective assessments that could be easily varied and swayed by individual examiners makes these screening mechanisms incomparable and unsatisfactory. Additionally, some non-nutritional factors such as inflammation, fluid status, renal dysfunction, and hepatic congestion also exert diverse effects on indicators like serum albumin and BMI (43, 44), effectively exposing these tools to additional noises. Thus, it is neither sufficient nor precise to evaluate patients' nutritional risk with regard to their cancer prognosis and treatment efficacy only by their BMI or albumin status.

Fortunately, NRI values measured by a combination of factors such as ideal body weight, serum albumin, and present body weight may overcome the shortcomings of individual indicators. In other words, creating patients' NRI score as a combined index of their ideal body weight, present body weight, and serum albumin levels has the potential to minimize the effects of fluid status, and in turn, distinguish nutritional risk better than individual indexes. As demonstrated in previous studies, one of the indexes under the NRI umbrella that could appraise forecasting risk of malnutrition-related incidence rate and mortality in advanced-age patients was the Geriatric Nutritional Risk Index (GNRI) (45). GNRI has been associated with poor treatment outcomes in many diseases, including cancer (46–50). Moreover, previous research also illustrated that in patients with new metastatic gastric adenocarcinoma and esophageal adenocarcinoma, pretreatment NRI and change of NRI in that were significant prognostic factors for OS.

Emerging evidence further suggests that evaluate NRI at baseline and during treatment can not only indicate patients' nutrition status but also provide useful prognostic information (51). Nevertheless, while meaningful insights are procurable, little is known about the association between NRI, prognosis, and treatment efficacy in breast cancer patients. To bridge the research gap, by analyzing the clinical and demographic attributes of 785 participants, our study demonstrated the clinical significance of using NRI to assess nutritional risk assessment in breast cancer patients. Our results indicated that high levels of NRI were significantly associated with more indicative clinicopathologic characteristics (age, menopause, US-LNM, total lymph nodes, and total axillary lymph nodes), nutritional parameters, and blood parameters (weight, BMI, ALT, AST, LDH, GGT, ALP, GLU, IgG, W, ALB, Hb, R, N, E, and P) of all breast cancer patients.

Through the univariate and multivariate Cox regression survival analyses, the preoperative NRI was an independent predictor of DFS and OS survive time. And the average DFS and OS survive time for patients who had high NRI scores were longer than for those who had low NRI scores by the log-rank analysis in the NACT group and the non-NACT group. Similar conclusions have been reached in many published studies focusing on other malignancies (52, 53). For instance, 143 patients with localized esophageal cancer treated with definitive concurrent chemoradiotherapy in a retrospective study conducted by Clavier and associates, multivariable analyses indicated that the NRI was an independent predictor for patients' overall survival (52). Moreover, Cox and colleagues retrospectively analyzed patients with esophageal cancer included chemoradiotherapy with or without cetuximab in the SCOPE1 clinical trial, reporting that NRI <100 in a baseline was significantly related to decreased overall survival in cancer patients (53).

Previous studies suggest that patients' NRI values were prognostic in a range of localized as well as metastatic tumors like esophageal cancer (54, 55). However, there is a dearth of research on the effects of NRI on prognosis and treatment efficacy in breast cancer patients. To bridge the research gap, we analyzed the relationship between pathologic stage and NRI in patients with breast cancer, and observed that patients who had high NRI scores had longer DFS and OS survive time than those who had low NRI values in both patients with early-stage breast cancer and advanced stage breast cancer. Furthermore, patients who had high NRI levels had longer DFS and OS survive time in contrast to those who had low NRI scores in molecular subtypes of breast cancer. Moreover, the results also performed the mean DFS and OS survive time in breast cancer patients who had high NRI scores were longer than in those patients who had low NRI scores with LVI status. Furthermore, we also analyzed the relationship between NRI and MPG/Response, and the results also shown that patients who had high NRI scores had longer DFS and OS survive time than those who had low NRI scores in different MPG grades, especially in MPG grade 2; and patients who had high NRI values had longer DFS and OS survive time in contrast to those who had low NRI scores in different responses.

All breast cancer patients could tolerate the neoadjuvant chemotherapy toxicities and adverse effects. The hematologic and gastrointestinal reactions were the common toxicities and adverse effects, and the results shown that there was no difference using the optimal NRI cutoff value of 112 in toxicity assessment, except in nausea, mouth ulcers, anemia, leukopenia, which should get doctors' as well as patients' attention. Using NRI as a prognostic marker and monitoring response to treatment make it possible to start timely interventions to reduce the risk of these complications.

As far as we know, this study is the first to illustrate the clinical and prognostic significance of NRI in a large cohort of breast cancer patients. Additionally, we also demonstrate that the change of NRI during treatment is a predictor for DFS and OS in different molecular subtypes and different lymph vessel invasion levels, as well as the relationship between NRI status and neoadjuvant chemotherapy toxicities.

However, the presented study is not without limitations. Firstly, our study evaluated the research topic from a retrospective perspective and was underway in a single-center with a relatively restricted number of breast cancer patients. To further enrich the literature, multicenter-based research that draws insights from large study populations should be encouraged. Secondly, as common in studies that adopt similar research methods (e.g., utilize eligibility criteria to screen patients), selection bias in our study could be difficult to eliminate. Thirdly, as NRI is a non-specific tumor marker, additional validation of the association between NRI, cancer prognosis, and treatment efficacy in large prospective studies should be conducted in the future.

## Conclusion

NRI is described as the significant predictor for breast cancer patients, and may forecast the survival and prognosis for breast cancer. The minimally invasive, easily accessible and convenient indicators should be help doctors in terms of selecting measures, evaluating the curative effect, and estimating the prognosis of breast cancer.

## Data Availability Statement

The raw data supporting the conclusions of this article will be made available by the authors, without undue reservation.

## Ethics Statement

This study was approved by the Ethics Committee of Cancer Hospital Chinese Academy of Medical Sciences and Tongji Hospital. The patients/participants provided their written informed consent to participate in this study.

## Author Contributions

LC and YQ: writing—original draft and writing—review & editing. XK and ZS: formal analysis. ZW, XW, and YD: data curation and investigation. YF and XL: methodology and supervision. XL and JW: resources, funding acquisition, and project administration. All authors contributed to the article and approved the submitted version.

## Funding

The work is partly supported by research grants from National Nature Science Foundation of China (Nos. 81872160, 82072940, 82103047, 82102887, and 81802676), Beijing Nature Science Foundation of China (Nos. 7191009 and 7204293), National Key Research and Development Program of China (No. 2018YFC1312100), China National Key Research and Development Program (Nos. 2020AAA0105000 and 2020AAA0105004), Special Research Fund for Central Universities, Peking Union Medical College (No. 3332019053), Beijing Hope Run Special Fund of Cancer Foundation of China (Nos. LC2020L01, LC2019B03, and LC2019L07), Wuhan Youth Cadre Project (Nos. 2017zqnlxr01 and 2017zqnlxr02), Clinical Research Physician Program of Tongji Medical College HUST (No. 5001540018), Golden Bridge Project Seed Fund of Beijing Association for Science and Technology (No. ZZ20004), Chinese Young Breast Experts Research project (No. CYBER-2021-005), 2021 Chaoyang District Social Development Science and Technology Plan Project (No. CYSF2115), Beijing Xisike Clinical Oncology Research Foundation (No. Y-Young2021-0017), and XianSheng Clinical Research Special Fund of China International Medical Foundation (No. Z-2014-06-2103).

## Conflict of Interest

The authors declare that the research was conducted in the absence of any commercial or financial relationships that could be construed as a potential conflict of interest.

## Publisher's Note

All claims expressed in this article are solely those of the authors and do not necessarily represent those of their affiliated organizations, or those of the publisher, the editors and the reviewers. Any product that may be evaluated in this article, or claim that may be made by its manufacturer, is not guaranteed or endorsed by the publisher.
